# PRMT5 inhibition promotes cross-species spermatogonia expansion and suppresses differentiation

**DOI:** 10.1186/s13619-026-00293-x

**Published:** 2026-07-07

**Authors:** Zhaokai Yao, Wen Wang, Kang Tang, Yuning Yang, Shaofang Ren, Yetian Weng, Linzi Ma, Dingyao Chen, Haoxu Gu, Jialin Wan, Zhaoting Liu, Yi Zheng, Chaohui Li, Fang Luo, Xiao-Yang Zhao, Yong Fan

**Affiliations:** 1 Department of Obstetrics and Gynecology, Guangdong Provincial Key Laboratory of Major Obstetric Diseases, Guangdong Provincial Clinical Research Center for Obstetrics and Gynecology, Guangdong-Hong Kong-Macao Greater Bay Area Higher Education Joint Laboratory of Maternal-Fetal Medicine, The Third Affiliated Hospital, Guangzhou Medical University, Guangzhou, 510150 China; 2 Department of Developmental Biology, School of Basic Medical Sciences, Southern Medical University, Guangzhou, China; 3 State Key Laboratory of Organ Failure Research, School of Laboratory Medicine and Biotechnology, Southern Medical University, Guangzhou, China; 4 Key Laboratory of Mental Health of the Ministry of Education, Guangzhou, China; 5 Guangdong Provincial Key Laboratory of Construction and Detection in Tissue Engineering, Southern Medical University, Guangzhou, Guangdong China; 6 Guangdong-Hong Kong Joint Laboratory for Psychiatric Disorders, Guangzhou, China; 7 Department of Gynecology, Zhujiang Hospital, Southern Medical University, Guangzhou, China; 8 National Clinical Research Center for Kidney Disease, Guangzhou, China

**Keywords:** Spermatogonial stem cells, PRMT5, EPZ015666, Male infertility, Cross-species validation

## Abstract

Spermatogonial stem cells (SSCs) hold great promise for treating male infertility, but their clinical translation is impeded by the lack of optimal conditions to maintain their undifferentiated state in vitro. In this study, we focused on epigenetic regulators upregulated during differentiation as potential targets. Through a small-molecule screen targeting such conserved regulators, we found that PRMT5 inhibition suppressed mouse SSC differentiation and enhanced their proliferation in a GDNF-deficient, differentiation-prone microenvironment in vitro. Using SSC transplantation assays, we confirmed that EPZ015666-treated SSCs retained their spermatogonial identity. This differentiation-inhibitory effect was reversible upon EPZ015666 withdrawal, allowing restoration of normal spermatogenesis. Notably, EPZ015666 also inhibited differentiation and promoted the proliferation of human and non-human primate spermatogonia in vitro. Mechanistically, EPZ015666 exerted this effect by inhibiting the enzymatic active site of PRMT5. These findings suggest that PRMT5 inhibition could provide a novel strategy for culturing human SSCs in vitro.

**Supplementary Information:**

The online version contains supplementary material available at 10.1186/s13619-026-00293-x.

## Background

Spermatogenesis is sustained by spermatogonial stem cells (SSCs), which maintain a dynamic balance between self-renewal and differentiation (Kanatsu-Shinohara and Shinohara 2013). This capacity not only supports lifelong sperm production but also makes SSCs themselves promising candidates for cell-based therapies to treat male infertility, a condition that affects over 100 million men worldwide. Potential applications include fertility restoration in prepubertal boys following gonadotoxic treatments, such as chemotherapy for childhood cancer (Brinster 2007). However, the development of SSC-based therapies faces a major technical barrier, which is the inability to achieve long-term expansion of human SSCs in vitro. Although methods for the long-term culture and transplantation of mouse SSCs are well established, the optimal conditions for the maintenance and propagation of human SSCs remain elusive.

Previous studies have identified the growth factors produced within the testicular microenvironment that support the formation and maintenance of mouse SSCs in vitro, which are designated germline stem cells (GSCs) (Kanatsu-Shinohara et al. 2003, 2005). For example, glial cell line–derived neurotrophic factor (GDNF) (Meng et al. 2000) and its downstream pathways are well-established as critical for SSC self-renewal in mice (Airaksinen And Saarma 2002; He et al. 2008; Lee et al. 2007; Naughton et al. 2006; Oatley et al. 2007). However, reliance on such cytokine-based paradigms has not yet enabled the long-term culture of human SSCs (Goharbakhsh et al. 2013; He et al. 2010; Sadri-Ardekani et al. 2009). Beyond extrinsic growth factors, the intrinsic balance between SSC self-renewal and differentiation is governed by precise molecular mechanisms. Crucially, the transition to differentiation is not merely a passive loss of stemness but an active process driven by specific molecular pathways. This is exemplified by pro-differentiation signals such as retinoic acid, which are essential for initiating differentiation (Bowles et al. 2006; Koubova et al. 2006). Consistent with this model, in vitro short-term cultured spermatogonia (SPG) from primates exhibited a molecular profile closely resembling that of in vivo differentiating progenitors and failed to maintain an undifferentiated SPG state (Bi et al. 2024). Importantly, this bias toward differentiation can be counteracted. For instance, inhibiting differentiation-promoting pathways such as the AKT pathway in human SPG enables expansion of the undifferentiated SPG in vitro (Tan et al. 2020). These findings suggest that actively suppressing differentiation-upregulated mechanisms may be a critical strategy for maintaining human SSCs in an undifferentiated state in vitro.

Notably, fate determination is precisely controlled by epigenetic mechanisms. Among these, DNA methyltransferases (e.g., DNMT3A/B) (Dura et al. 2022; Shirakawa et al. 2013) and histone modifiers (e.g., KMT2B, SETDB1, DOT1L) (An et al. 2014; Lambrot et al. 2015; Li et al. 2020; Lin et al. 2022; Tomizawa et al. 2018) have been shown to be crucial for SSC maintenance and differentiation in model systems. However, whether targeting these epigenetic regulators can establish a stable human SSC culture system remains largely unexplored.

In this study, we observed upregulation of several DNA and histone methyltransferases in differentiating SPG. Through small-molecule screening targeting these epigenetic modifiers, we identified that EPZ015666, an inhibitor of protein arginine methyltransferase 5 (PRMT5), could sustain cultured mouse SSCs proliferation in the absence of GDNF while inhibiting spermatogonial differentiation. Furthermore, mouse SSCs expanded with EPZ015666 successfully engrafted into seminiferous tubules upon transplantation, restored spermatogenesis, and ultimately generated functional sperm cells. Notably, we demonstrated that EPZ015666 could support the in vitro culture of undifferentiated SPG from both human and non-human primates. Collectively, our findings provide a strategy with significant implications for culturing and expanding human SPGs for future clinical use.

## Results

### A screen identifies the PRMT5 inhibitor EPZ for mouse SSC maintenance

To uncover novel epigenetic factors that regulate SSC fate, we analyzed single-cell transcriptomes from human, macaque, and mouse (Shami et al. 2020). Consistent with previous reports, expression patterns of conserved marker genes suggested that SPG1-2 represent undifferentiated SPG, SPG3-5 correspond to progressive differentiation stages, and SPG6 consists of cells preparing for meiosis (Fig. S1A, B). Our analysis revealed that the transition from undifferentiated (SPG1-2) to differentiating (SPG3-5) SPG was accompanied by the upregulation of epigenetic modifiers, particularly DNA and histone methyltransferases such as *DNMT1, EHMT2, EZH2, PRMT3* and *PRMT5* (Fig. S1C). Furthermore, the gene scores of “Histone H4R3 Methylation” and “Maintenance of DNA Methylation” showed higher levels in differentiating SPG (SPG3–5) than in undifferentiated SPG (SPG1–2) (Fig. S1D). These findings led us to hypothesize that these upregulated epigenetic regulators might be required for spermatogonial differentiation. If so, inhibiting their activity would suppress SPG differentiation and maintain them in a proliferative and undifferentiated state.

**Fig. 1:**
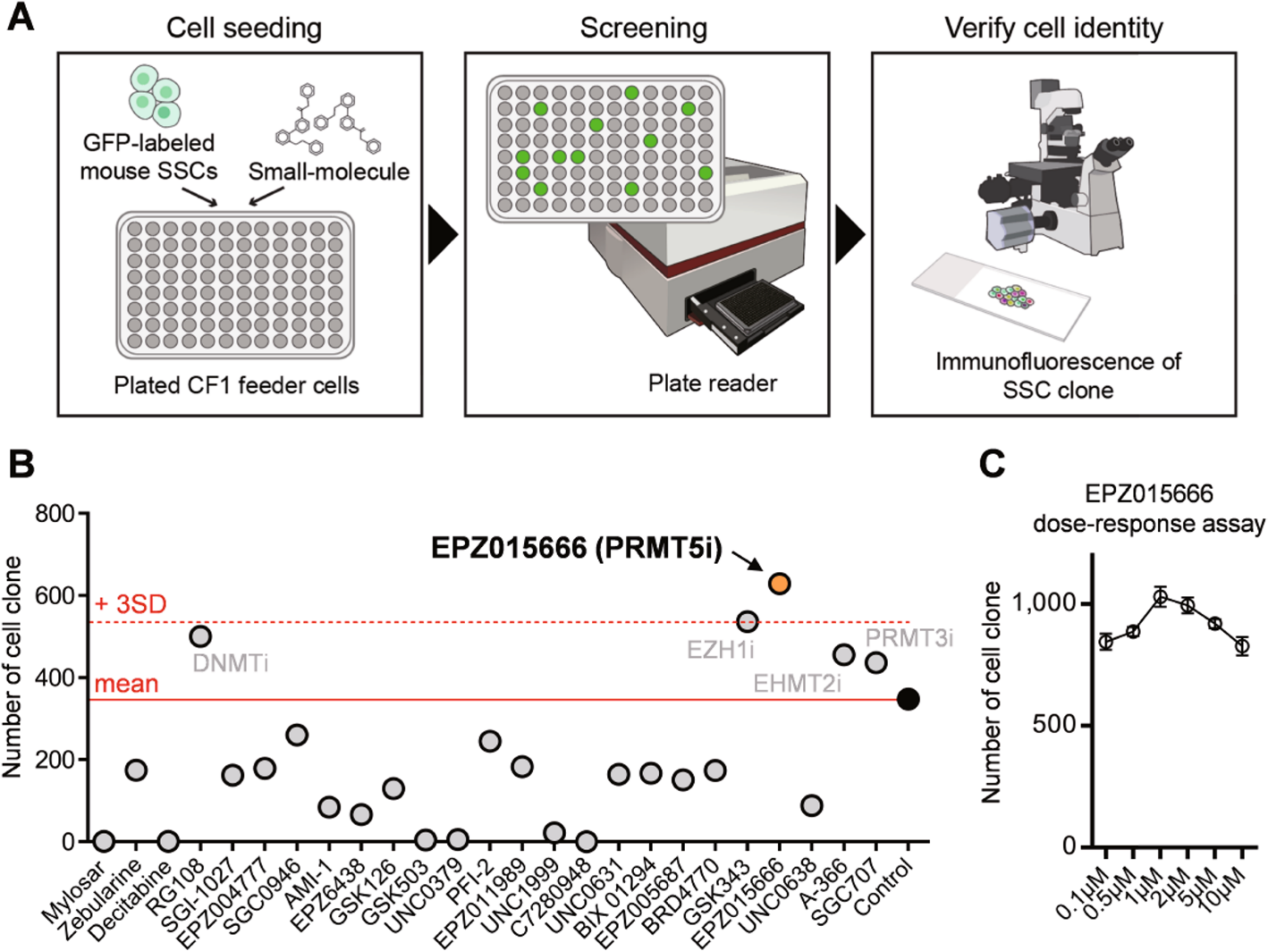
Screening for small molecule inhibitors. **A** Schematic outline of the small-molecule screening. **B** Scatter-plot representation of the results of chemical library screening (10 μM). The average value (red line) and 3 SDs (standard deviations: red dotted lines) for the negative controls are indicated. **C** EPZ015666 concentration gradient test (*n* = 3 biologically independent samples). Error bars indicate mean ± SEM from three independent experiments

To test this, we conducted a small-molecule screen targeting these factors under conditions that induce spermatogonial differentiation (Fig. 1A). As a negative control (NTC), we used GDNF withdrawal, which prior reports indicate triggers early SSC differentiation (Brinster 2007; Kubota et al. 2004a,b; Ryu et al. 2005). This approach aimed to identify compounds that could suppress differentiation and thereby promote cultured mouse SSCs expansion over 6 days. Our primary screen identified several hits corresponding to inhibitors of genes up-regulated during SPG differentiation. This finding largely supports our hypothesis (Fig. 1B, Fig. S1C, Table 1). Among these, the PRMT5 inhibitor EPZ015666 (hereafter EPZ) exhibited the most potent effect, increasing the number of cell clones by more than 3 standard deviations above the mean of NTC replicates (Fig. 1B). A dose–response assay based on cell clone number confirmed its efficacy and identified 1 μM as the optimal concentration for maximizing clone formation. This concentration was used in all subsequent culture experiments (Fig. 1C).

**Table 1:** List of small-molecule compounds for screening

MOLENAME	CAS	Receptor
Mylosar	320-67−2	DNA methyltransferase (DNMT)
Zebularine	3690-10-6	Cytidine deaminase
Decitabine	2353-33−5	DNA methyltransferase (DNMT)
RG108	48208-26−0	DNA methyltransferase (DNMT)
SGI-1027	1020149-73−8	DNMT1; DNMT3A; DNMT3B
EPZ004777	1338466-77−5	DOT1L
SGC0946	1561178-17−3	DOT1L
AMI-1	20324-87−2	PRMT1
EPZ6438	1403254-99−8	EZH2
GSK126	1346574-57−9	EZH2
GSK503	1346572-63−1	EZH2
UNC0379	1620401-82−2	SETD8
PFI-2	1627676-59−8	SETD7
EPZ011989	1598383-40−4	EZH2
UNC1999	1431612-23−5	EZH1; EZH2
C7280948	587850-67−7	PRMT1
UNC0631	1320288-19−4	EHMT2
BIX 01294	1392399-03−9	EHMT2
EPZ005687	1396772-26−1	EZH2
BRD4770	1374601-40−7	EHMT2
GSK343	1346704-33−3	EZH1; EZH2
EPZ015666	1616391-65−1	PRMT5
UNC0638	1255580-76−7	EHMT2
A-366	1527503-11−2	EHMT2
SGC707	1687736-54−4	PRMT3

### EPZ treatment promotes spermatogonia expansion while suppressing their differentiation

**Fig. 2:**
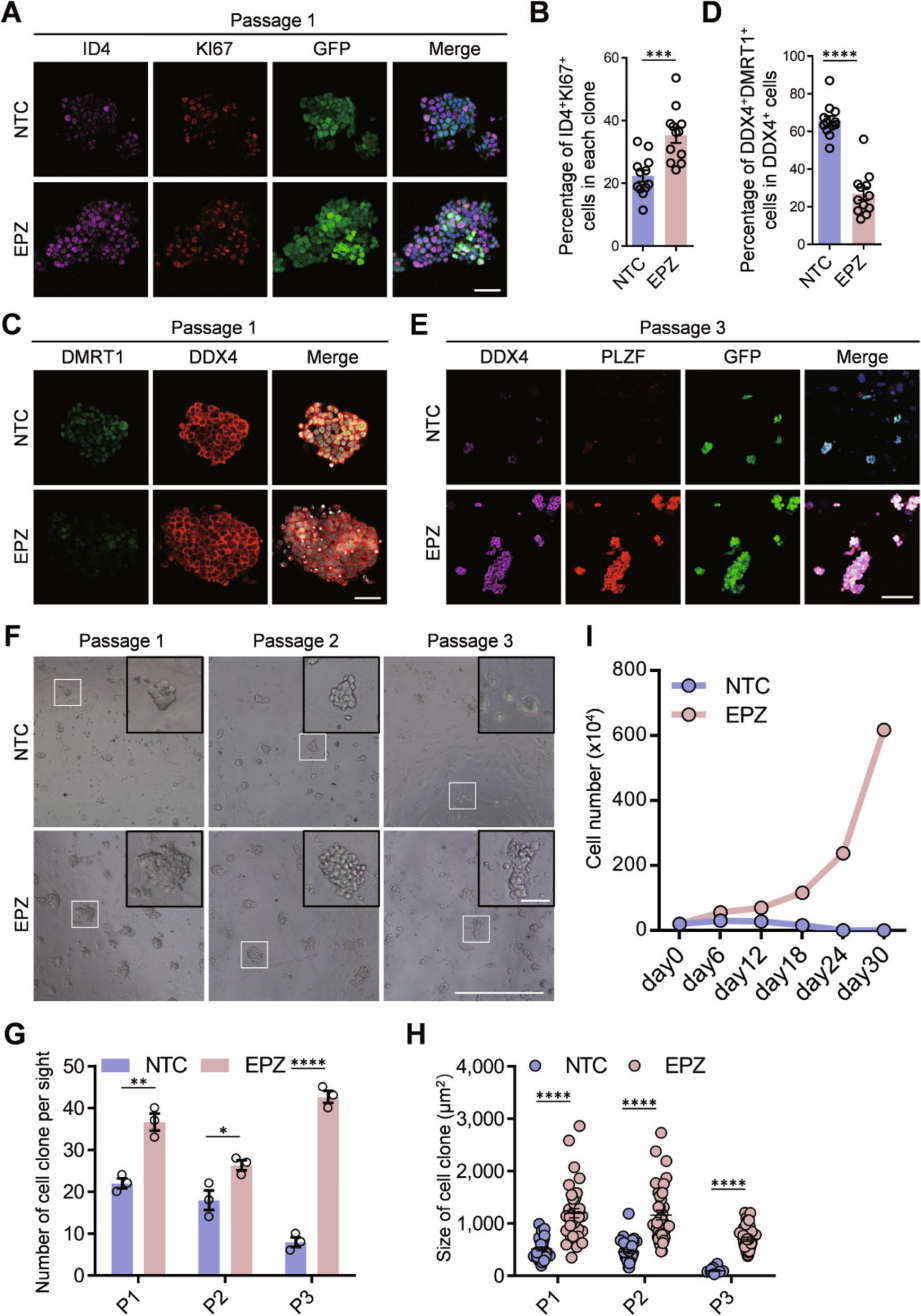
EPZ treatment promotes spermatogonia expansion and inhibits differentiation. **A** Immunofluorescent staining of ID4^+^KI67^+^ cells cultured in NTC and EPZ groups. Scale bar, 50 μm. **B** Quantitative analysis of the percentage of ID4^+^KI67^+^ cells in each clone. Each circle represents the statistical result from one clone (12 clones from each group; *n* = 3 biologically independent samples). Data are presented as the mean ± SEM; unpaired two-sided Student’s t-test; ***P < 0.001. **C** Immunofluorescent staining of DMRT1^+^DDX4^+^ cells in NTC and EPZ groups using SSCs from B6D2F1 male mice (C57BL/6 × DBA/2). Scale bar, 50 μm. **D** Quantitative analysis of the percentage of DMRT1^+^DDX4^+^ cells in DDX4^+^ cells. Each circle represents the statistical result from one clone (11 clones from each group;* n* = 3 biologically independent samples). Data are presented as the mean ± SEM; unpaired two-sided Student’s t-test; ****P < 0.0001. **E** Immunofluorescence analysis of DDX4^+^PLZF^+^ cells in NTC and EPZ groups. Scale bar, 50 μm. **F** Bright-field images of cultured mouse SSC clones over three passages. The black-bordered image shows a magnified view of the area within the white square. Scale bars: 250 μm and 50 μm (magnified view). **G** Quantitative analysis of the number of cell clones per sight from Fig. 2F. Each circle represents the statistical result from one field of view (*n* = 3 biologically independent samples). Data are presented as the mean ± SEM; unpaired two-sided Student’s t-test; *P < 0.05, **P < 0.01, ****P < 0.0001. **H** Quantitative analysis of the clone size from Fig. 2F. Each circle represents the statistical result from one clone (*n* = 3 biologically independent samples). Data are presented as the mean ± SEM; unpaired two-sided Student’s t-test; ****P < 0.0001. **I** SSC proliferation curves in NTC and EPZ groups across five passages (up to Day 30)

To validate cell identity, we performed immunofluorescence analysis. In the first passage, we observed a significantly higher number of cells that were double-positive for the mouse SSC marker ID4 (Chan et al. 2014; Sun et al. 2015) and the proliferation marker KI67 in the EPZ group compared with the NTC group (Fig. 2A, B). Furthermore, immunofluorescence analysis showed that the EPZ group had significantly fewer DDX4^+^DMRT1^+^ cells than the NTC group (Fig. 2C, D), while DMRT1 is known to promote spermatogonial differentiation in SPG (Matson et al. 2010; Zhang and Zarkower 2017).

To evaluate the effects of EPZ treatment on cultured mouse SSCs over multiple passages, we compared the EPZ group with the NTC group. By the third passage (day 18), the EPZ group maintained distinct expression of the key undifferentiated SPG marker PLZF (Buaas et al. 2004; Costoya et al. 2004), whereas PLZF expression was nearly absent in the NTC group (Fig. 2E). Furthermore, during three consecutive passages, the EPZ group consistently exhibited notably larger clones and a significantly higher clone count than the NTC group. In contrast, the NTC group exhibited a progressive decline in both clone size and number, eventually losing typical SSC clonal morphology (Fig. 2F-H). Notably, by the fifth passage (day 30), a gradual increase in cell number was still observed in the EPZ group, whereas the NTC group had almost diminished (Fig. 2I).

To determine the in vivo impact of EPZ, we treated mice with EPZ, using the solvent vehicle as the control (Fig. S2A). In comparison with the vehicle group, the testes from EPZ-treated mice showed a significant increase in PLZF^+^ undifferentiated SPG (Fig. S2B). Conversely, the proportion of KIT^+^ differentiating SPG (Schrans-Stassen et al. 1999) was markedly reduced in the EPZ group (Fig. S2C). These in vivo findings further support that EPZ treatment promotes the expansion of undifferentiated SPG while suppressing their differentiation.

### The inhibition of spermatogonial differentiation by EPZ is reversible

To validate the effect of EPZ within a more physiologically relevant context, we utilized a mouse testicular tissue culture system (Sato et al. 2011). After 7 days of culture, compared with the DMSO group, EPZ-treated tissues exhibited a significant increase in the percentage of ID4^+^ SSCs among DDX4^+^ cells and a decrease in the percentage of STRA8^+^ early primary spermatocytes among DDX4^+^ cells (Nie et al. 2022) (Fig. S3A-E).

**Fig. 3:**
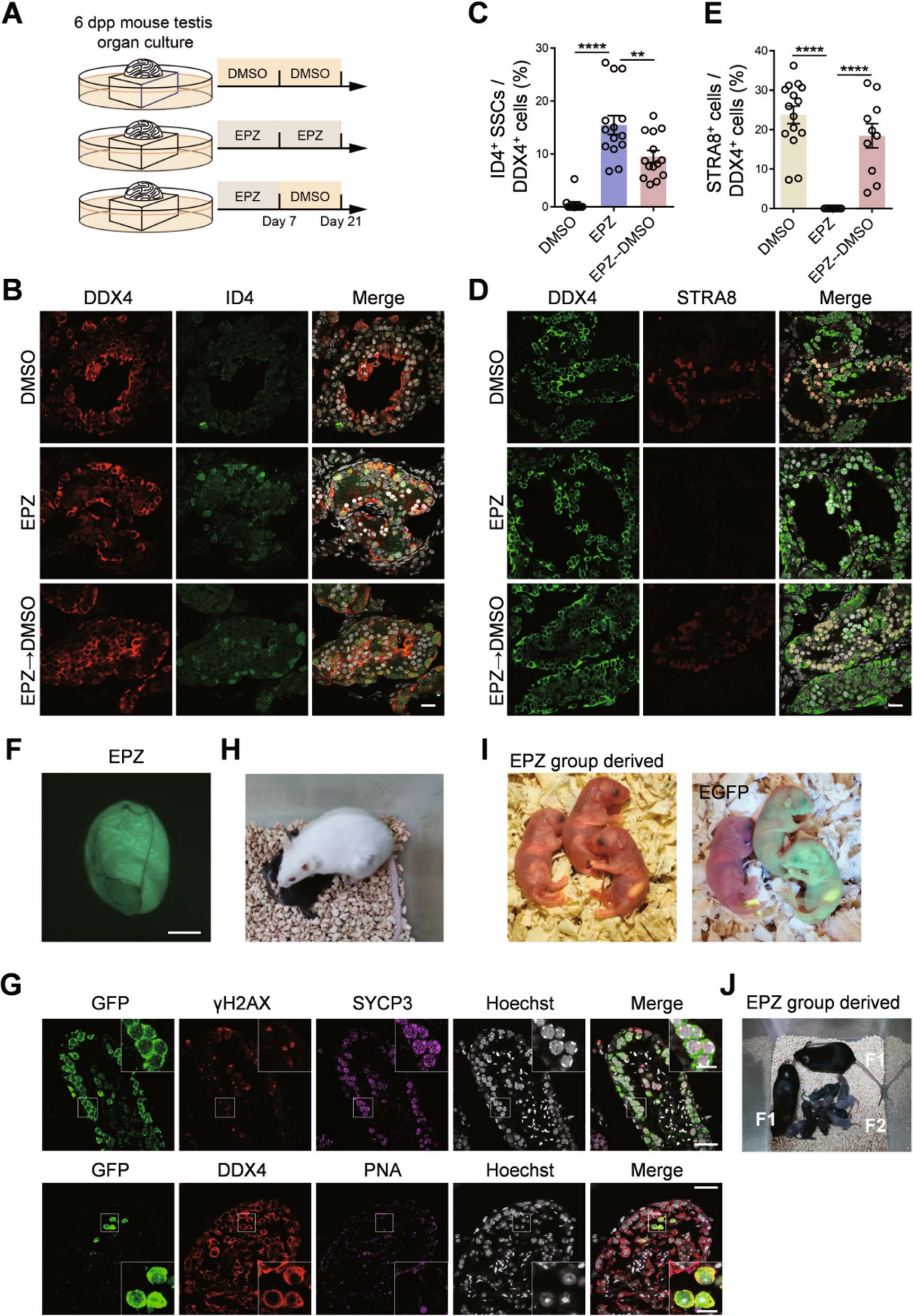
The inhibition of SSC differentiation by EPZ is reversible. **A** Schematic for the 6 dpp mouse testes organ culture. Mouse testis treated with DMSO or EPZ at day 21. Additionally, another group received EPZ for 7 days, then DMSO for 14 days (EPZ → DMSO). **B** Immunofluorescence of DDX4^+^ID4^+^ cells in a section of mouse testis by in vitro culture at day 21. Scale bar, 20 μm. **C** Quantitative analysis of the percentage of ID4^+^ SSCs among DDX4^+^ cells in each group. Each circle represents the statistical result from one tissue Sect. (12, 14, 14 sections from left to right; *n* = 3 biologically independent samples). Data are presented as the mean ± SEM; unpaired two-sided Student’s t-test; **P < 0.01, ****P < 0.0001. **D** Immunofluorescence of STRA8^+^DDX4^+^ cells in a section of mouse testis by in vitro culture at day 21. Scale bar, 20 μm. **E** Quantitative analysis of the percentage of STRA8^+^ cells among DDX4^+^ cells in each group. Each circle represents the statistical result from one tissue Sect. (10 sections from each group; *n* = 3 biologically independent samples). Data are presented as the mean ± SEM; unpaired two-sided Student’s t-test; ****P < 0.0001. **F** Cell colonization in the recipient testes by transplantation assay. Scale bar, 1 mm. **G** Immunofluorescent staining of γH2AX^+^SYCP3^+^ cells (up) and DDX4^+^PNA^+^ cells (down) in recipient testes that were transplanted with GFP-labeled SSCs with EPZ-treatment after 10 passages. The white box indicates the magnified area. Scale bars, 50 μm and 10 μm (magnified view). **H** Pseudopregnant mice (with an ICR background) gave birth to offspring from the EPZ-treated group. **I** The offspring derived from spermatozoa from the EPZ group. **J** The offspring were fertile from the EPZ group

We next asked whether the differentiation-inhibitory effect of EPZ was reversible, a critical feature for its potential use as a controllable tool (Hou et al. 2013; Li et al. 2013). To test this, testicular tissue cultures were treated with EPZ or DMSO for 7 days, followed by a 14-day culture period under three conditions: continuous DMSO (DMSO group), continuous EPZ (EPZ group), or EPZ withdrawal and replaced by DMSO (EPZ → DMSO group) (Fig. 3A). Immunofluorescence staining revealed that in the EPZ group, the percentage of ID4^+^ SSCs among DDX4^+^ cells was significantly increased compared with the DMSO group (Fig. 3B, C), whereas the percentage of STRA8^+^ early primary spermatocytes among DDX4^+^ cells was notably reduced and barely detectable (Fig. 3D, E). Strikingly, upon EPZ withdrawal, this pattern was reversed. By day 21, the percentage of ID4^+^ SSCs among DDX4^+^ cells in the EPZ → DMSO group was markedly reduced (Fig. 3B, C), while the percentage of STRA8^+^ early primary spermatocytes among DDX4^+^ cells was significantly increased compared with the EPZ group (Fig. 3D, E). These findings demonstrate that the EPZ-mediated effects on SSCs could be reversed upon its withdrawal.

### Mouse SSCs expanded through EPZ treatment retain functional competence

To further assess whether EPZ treatment compromises the functional competence of SSCs, we performed transplantation assays. GFP-labeled SSCs from B6D2F1 male mice (EGFP-C57BL/6 × DBA/2) were cultured with EPZ for 10 passages (day 60) (Kanatsu-Shinohara et al. 2003). Subsequently, these cells were transplanted into the seminiferous tubules of infertile mice that had been induced by busulfan treatment (Brinster et al. 2003). Two months after transplantation, EPZ-treated cultured mouse SSCs were observed colonizing the seminiferous tubules of the recipient mice (Fig. 3F). Additionally, following EPZ treatment, cultured mouse SSCs retained their differentiation potential. This was evidenced by the presence of GFP-positive cells across various germ cell stages, including DDX4^+^PLZF^+^ undifferentiated SPG, SYCP3^+^γH2AX^+^ spermatocytes (Eijpe et al. 2000; Mahadevaiah et al. 2001), and DDX4^+^PNA^+^ spermatids (Fig. 3G, S3F). To test the fertility of the sperm produced, GFP-positive spermatozoa were isolated from the transplanted recipient mice and microinjected into oocytes. In total, 14 embryos were produced, and 12 two-cell embryos were transferred into the oviducts of pseudopregnant mice 24 h after sterile mating with vasectomized males. The pseudopregnant mice gave birth to 3 pups naturally (Fig. 3H, I, Fig. S3G) and the offspring were fertile and produced progeny (Fig. 3J). Together, these functional assays demonstrate that cultured mouse SSCs expanded through EPZ treatment retain their developmental potential, including the capacity to undergo spermatogenesis and generate fertile offspring.

### EPZ maintains undifferentiated SPG numbers under spermatogenesis-defective conditions

**Fig. 4:**
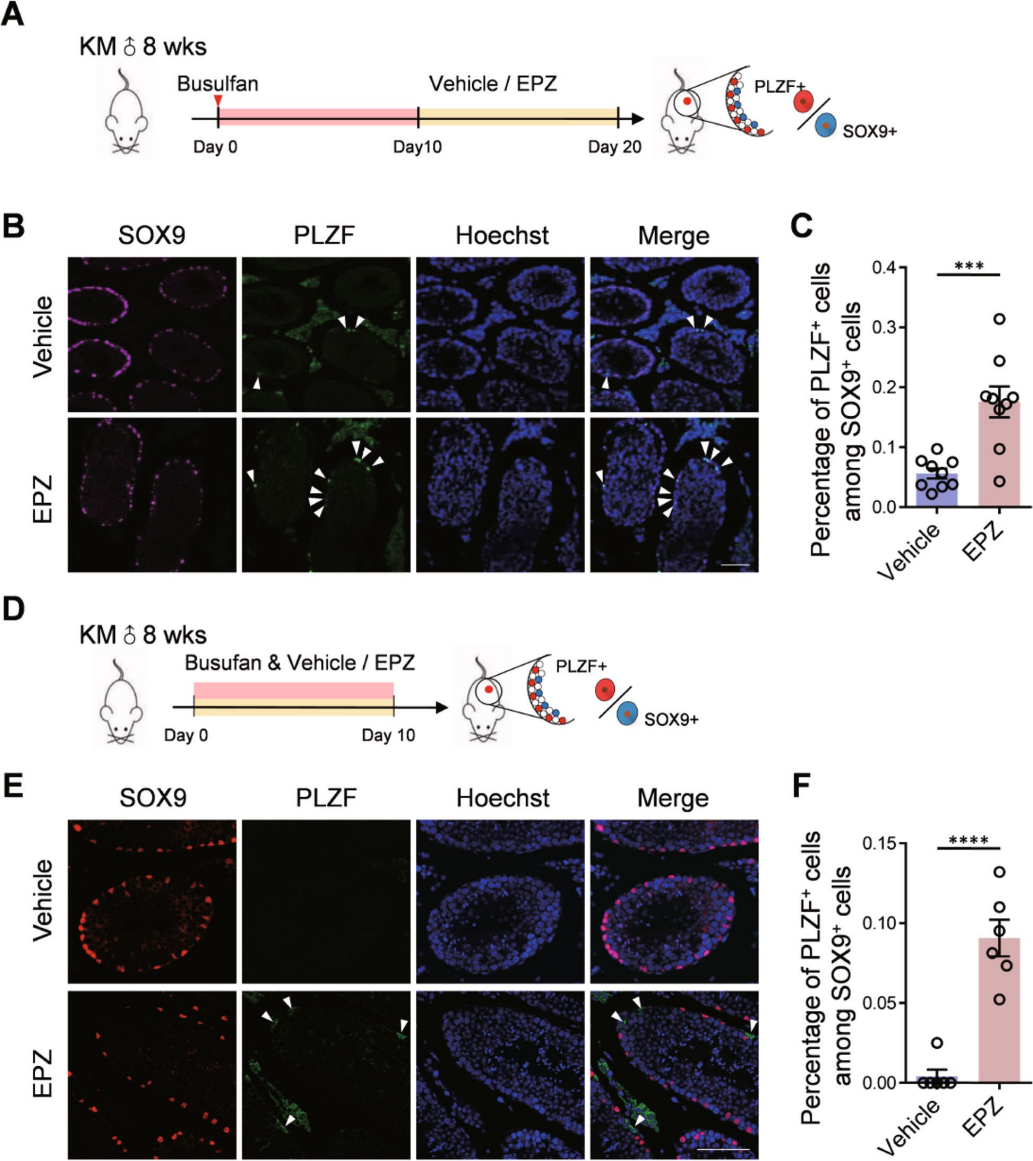
EPZ maintains undifferentiated SPG numbers under spermatogenesis-defective conditions. **A** Schematic outline of EPZ injection after 10 days of busulfan treatment in order to simulate reparation after chemotherapy. **B** Immunofluorescent staining of PLZF^+^ undifferentiated SPG and SOX9^+^ Sertoli cell in mouse testis under the treatment of busulfan with EPZ or Vehicle injection. The white arrow indicates PLZF^+^ cells. Scale bar, 50 μm. **C** Quantitative analysis of the percentage of PLZF^+^ cells among Sox9^+^ cells. Each circle represents the statistical result from one tissue Sect. (9 sections from each group; *n* = 2 biologically independent samples). Data are presented as the mean ± SEM; unpaired two-sided Student’s t-test; ***P < 0.001. **D** Schematic outline of mouse treated by busulfan simultaneously with EPZ injection in order to assess the prevention of chemotherapy injury. **E** Immunofluorescent staining of PLZF^+^ undifferentiated SPG and SOX9^+^ Sertoli cell in mouse testis under the treatment of busulfan with EPZ or Vehicle injection. The white arrow indicates PLZF^+^ cells. Scale bar, 50 μm. **F** Quantitative analysis of the percentage of PLZF^+^ cells among Sox9^+^ cells. Each circle represents the statistical result from one tissue Sect. (6 sections from each group; *n* = 2 biologically independent samples). Data are presented as the mean ± SEM; unpaired two-sided Student’s t-test; ****P < 0.0001

Previous studies have demonstrated that busulfan chemotherapy induces extensive apoptosis in undifferentiated SPG (Liu et al. 2014; Marcon et al. 2010; Zohni et al. 2012), accompanied by functional impairments in the niche-supporting Sertoli cells and Leydig cells, in which the decrease of GDNF was observed (Brilhante et al. 2012; Sasso-Cerri et al. 2017). Given EPZ’s capacity to promote proliferation of undifferentiated SPG without GDNF, we examined its effect under chemotherapy-induced conditions (La et al. 2022). Previous studies have confirmed that administration of busulfan (10 mg/kg) to adult mice induces a marked depletion of undifferentiated SPG by day 10 post-treatment, which triggers a regenerative response in the residual SPG, followed by a recovery to maximal numbers by day 20 (Kitadate et al. 2019; La et al. 2022). Therefore, to assess EPZ’s effect on this regenerative process, adult mice were treated with EPZ or an equal volume of vehicle on day 10 after busulfan administration (10 mg/kg) (Fig. 4A). The effect of EPZ on spermatogonial regeneration was then assessed on day 20 post-busulfan. At this assessment time point (day 20), the EPZ group exhibited a significant increase in PLZF^+^ undifferentiated SPG compared with the vehicle group (Fig. 4B, C).

We next asked whether EPZ administration simultaneously with busulfan treatment would affect spermatogonial numbers. To test this, we administered EPZ or vehicle simultaneously with busulfan treatment (Fig. 4D). We found that by day 10 post treatment, the percentage of PLZF^+^ undifferentiated SPG among Sox9^+^ Sertoli cells was higher in the EPZ group than in the vehicle group (Fig. 4E, F).

### PRMT5 promotes spermatogonial differentiation via its enzymatic activity

To investigate whether PRMT5 is the primary target mediating the effects of EPZ, we first asked if genetic perturbation of PRMT5 could mimic EPZ treatment (Chan-Penebre et al. 2015). Transient knockdown of *Prmt5* in cultured mouse SSCs using siRNA (Fig. S4A, B) under GDNF-free conditions significantly reduced the expression levels of DMRT1 (Fig. S4C).

**Fig. 5:**
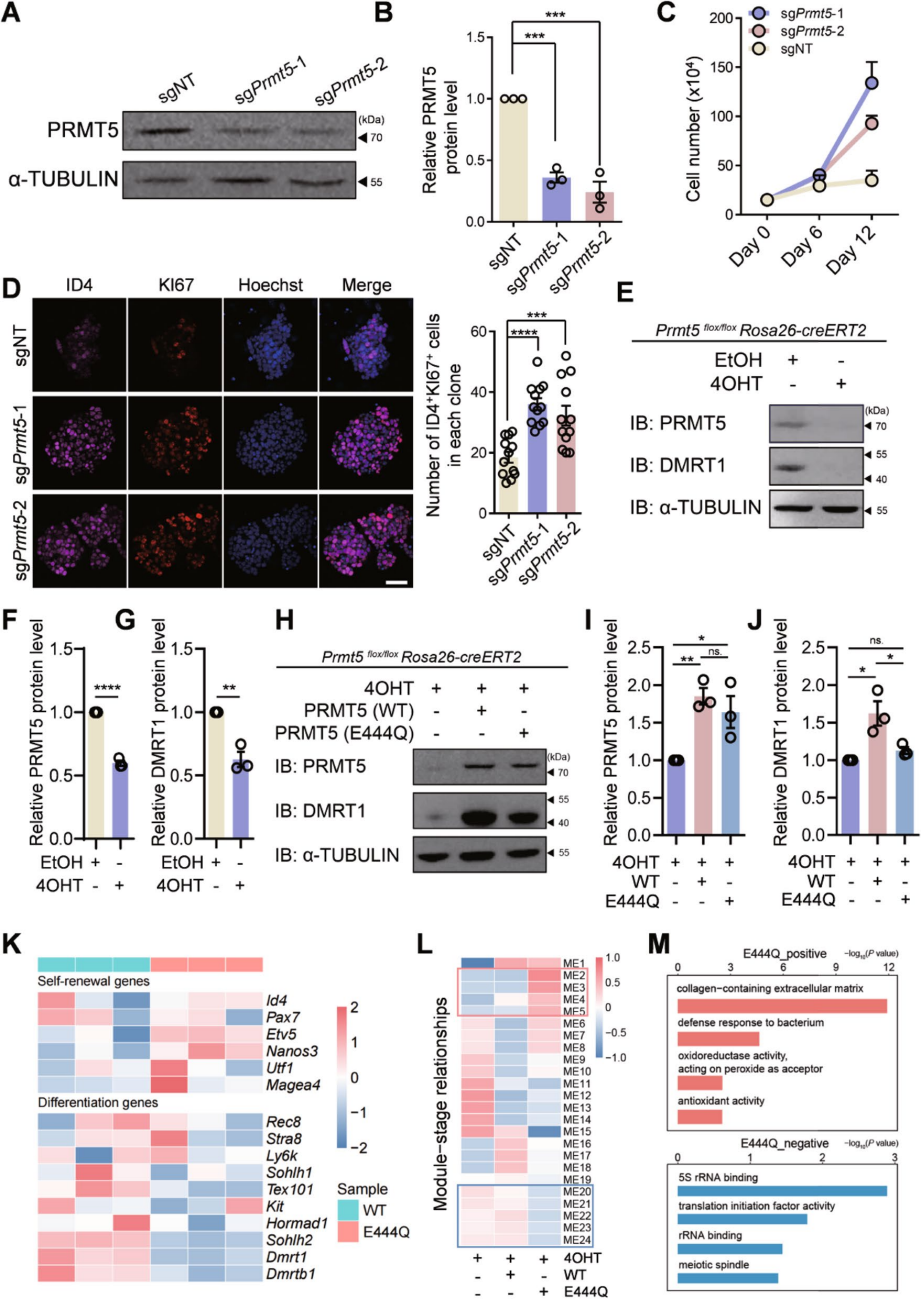
PRMT5 promotes the SSC differentiation via its enzymatic activity. **A** Western blot of *Prmt5* knockdown mediated by CRISPR-Cas13d system. sg*Prmt5*−1 and sg*Prmt5*−2 were designed to knock down *Prmt5*, while sgNT indicates non-targeting sgRNA. α-Tubulin was used as the loading control. **B** Quantitative analysis of relative PRMT5 protein level in (A) from three independent experiments. Error bars indicate mean ± SEM; unpaired two-sided Student’s t-test; ***p < 0.001. **C** Cell growth curve of in vitro cultured mouse SSCs. Error bars indicate mean ± SEM (*n* = 3 biologically independent samples). **D** Immunofluorescent staining of ID4^+^KI67^+^ cultured mouse SSCs in different groups for 18 days (left). Scale bar, 50 μm. Quantitative analysis of the number of ID4^+^KI67^+^ cells in each clone (right). Each circle represents the statistical result from one clone (12 views from each group; *n* = 3 biologically independent samples). Data are presented as the mean ± SEM; unpaired two-sided Student’s t-test; ***P < 0.001, ****P < 0.0001. **E** The expression of PRMT5 and DMRT1 was analyzed by western blotting in *Prmt5 *^*flox/flox*^*; Rosa26-creERT2* cultured mouse SSCs treated with ethanol (EtOH) or 4-hydroxytamoxifen (4OHT). **F** Quantitative analysis of relative PRMT5 protein level in (E) from three independent experiments. Error bars indicate mean ± SEM; unpaired two-sided Student’s t-test; ****P < 0.0001. **G** Quantitative analysis of relative DMRT1 protein level in (E) from three independent experiments. Error bars indicate mean ± SEM; unpaired two-sided Student’s t-test; **P < 0.01. **H** The expression of PRMT5 and DMRT1 was analyzed by western blotting in *Prmt5 *^*flox/flox*^*; Rosa26-creERT2* cultured mouse SSCs treated with 4OHT and transfected with lentivirus-mediated PRMT5 (WT) or PRMT5 (E444Q). **I** Quantitative analysis of relative PRMT5 protein level in (H) from three independent experiments. Error bars indicate mean ± SEM; unpaired two-sided Student’s t-test; ns, not significant, *P < 0.05, **P < 0.01. **J** Quantitative analysis of relative DMRT1 protein level in (H) from three independent experiments. Error bars indicate mean ± SEM; unpaired two-sided Student’s t-test; ns, not significant, *P < 0.05. **K** Heatmap of some classical SSC self-renewal and differentiation genes. The color key from blue to red indicates low (blue) to high (red) expression. **L** The heatmap represents the module-stage relationships between module eigengenes. **M** GO analysis of genes in PRMT5 E444Q_positive modules and PRMT5 E444Q_negative modules. *P* values were calculated by one-tailed hypergeometric test

For extended analysis, we established a stable *Prmt5* knockdown in cultured mouse SSCs using CRISPR-Cas13d (Fig. 5A, B). Compared with the control group, stable *Prmt5* knockdown notably increased the proliferation rate of cultured mouse SSCs under GDNF-free conditions (Fig. 5C). Consistently, the number of ID4^+^KI67^+^ proliferating SSCs was significantly higher in *Prmt5* knockdown groups than in the control group (Fig. 5D). Accordingly, the genes involved in SPG differentiation (*Stat3*, *Dmrt1* and *Kit*) were significantly down-regulated in *Prmt5* knockdown groups (Fig. S4D). These results suggest that knockdown of *Prmt5* essentially recapitulates the key features induced by EPZ treatment.

As EPZ inhibits the enzymatic activity of PRMT5 (Chan-Penebre et al. 2015), we next asked whether this catalytic activity is required for PRMT5’s role in cultured mouse SSCs. To address this, we generated *Prmt5*-knockout cultured mouse SSCs from *Prmt5 *^*flox/flox*^; *Rosa26-CreERT2* mice by treating them with 1 µM 4-hydroxytamoxifen (4OHT) in vitro (Dong et al. 2021). Compared with the ethanol-treated (EtOH) control group, the expression level of PRMT5 and DMRT1 dramatically was reduced in these knockout SSCs treated with 4OHT (Fig. 5E-G). In this knockout background, we performed a rescue experiment by transfecting either wild-type PRMT5 (WT) or a catalytically dead PRMT5 mutant (E444Q) (Antonysamy 2017) into the 4OHT-treated *Prmt5*-knockout SSCs. In the rescue experiments, overexpression of either PRMT5 (WT) or PRMT5 (E444Q) restored PRMT5 expression levels (Fig. 5H, I). Remarkably, overexpression of PRMT5 (WT) restored DMRT1 expression, whereas the catalytically dead PRMT5 (E444Q) mutant failed to do so (Fig. 5H, J). Bulk RNA-seq was further conducted to compare the gene expression profiles of the 4OHT-treated *Prmt5*-knockout SSCs overexpressing PRMT5 (WT) or PRMT5 (E444Q). Specifically, the overexpression of PRMT5 (E444Q) group downregulated the expression of multiple markers of spermatogonial differentiation genes, including *Kit*, *Dmrt1*, *Sohlh1*, and *Sohlh2* (Fig. 5K). To discover important molecular events, weighted gene co-expression network analysis (WGCNA) was performed on the transcriptome data, which yielded 24 gene modules (Fig. 5L). We then focused on the modules that were significantly correlated with the 4OHT-treated *Prmt5*-knockout SSCs overexpressing PRMT5 (E444Q), including four positives and five negatives (Fig. 5L). Notably, the negatively correlated modules were significantly enriched in meiotic spindle (Fig. 5M). Collectively, these findings suggest that EPZ likely inhibits spermatogonial differentiation by targeting PRMT5, and that PRMT5 promotes differentiation in a manner dependent on its methyltransferase activity.

### EPZ maintains undifferentiated SPG and suppresses their differentiation in monkeys and humans

Considering that *PRMT5* is consistently upregulated in differentiating SPG across species (Fig. S1) and that its inhibition with EPZ could expand cultured mouse SSCs, we next sought to preliminarily assess whether EPZ could exert similar effects in non-human primates and humans. To this end, we first enriched SPG from monkey and human testicular biopsies using magnetic-activated cell sorting (MACS) for the conserved surface marker ITGA6 (Maki et al. 2009; Shinohara et al. 1999; Valli et al. 2014). Immunofluorescence staining revealed that approximately 40% of the sorted ITGA6^+^ cells from monkey testes were UTF1^+^DDX4^+^ undifferentiated SPG, while those from human testes were UCHL1^+^ID4^+^ undifferentiated SPG (Fig. S5A, B). This method achieved a higher purity than previously reported methods (Medrano et al. 2016). In contrast, the ITGA6^−^ fraction from both species showed no detectable undifferentiated SPG (Fig. S5A, B).

**Fig. 6:**
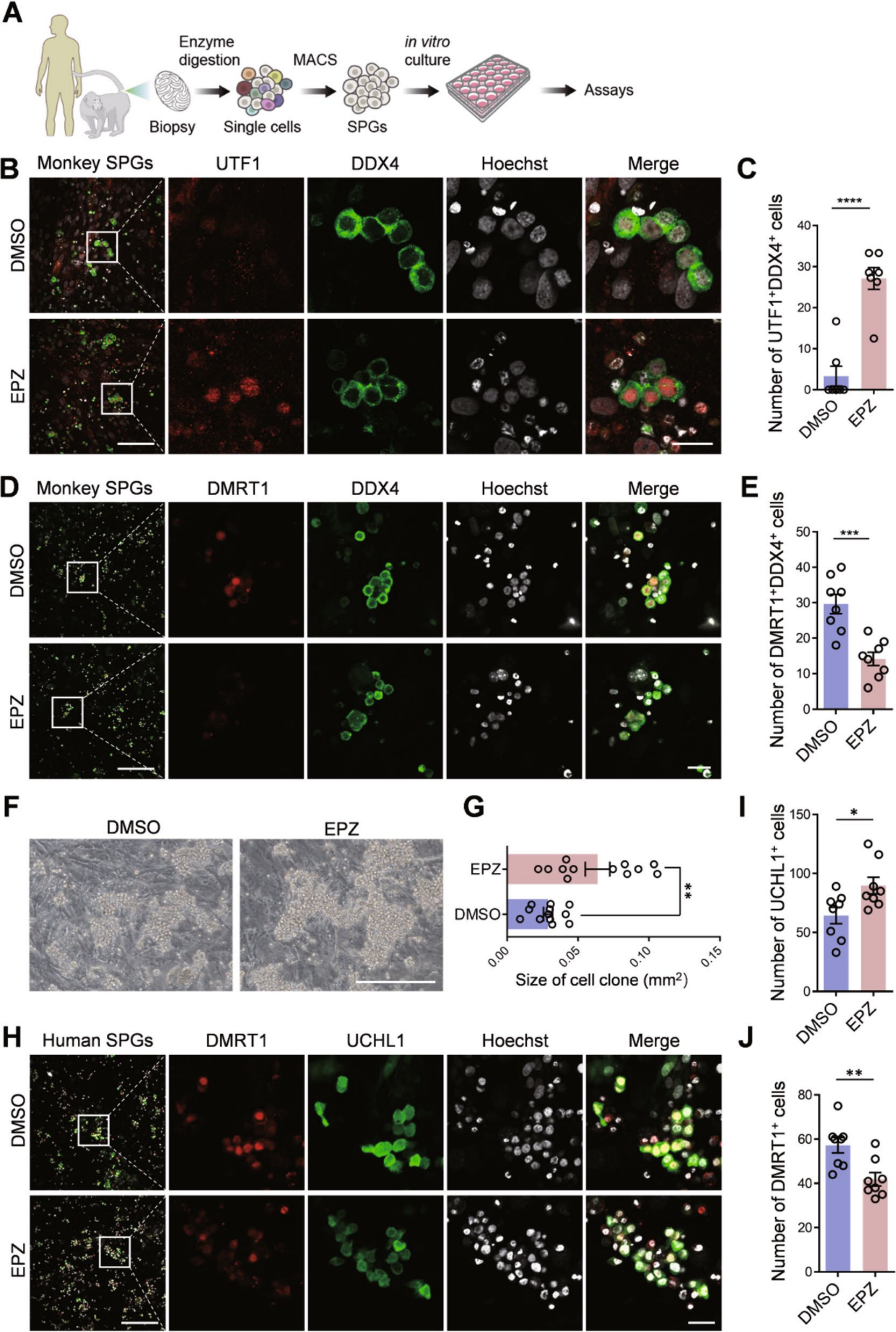
Cross-Species SSC maintenance by EPZ in vitro. **A** Schematic illustration of the human and monkey SPG culture experimental workflow. **B** Immunofluorescence of UTF1^+^DDX4^+^ cells in monkey cultured SPG. The right panel shows the clone enlarged in the white dashed box of the left field. Scale bars: 100 μm (left) and 10 μm (right). **C** Quantitative analysis of the number of UTF1^+^DDX4^+^ cells in each group. Each circle represents the statistical result from one field of view (7 views from each group; *n* = 2 biologically independent samples). Data are presented as the mean ± SEM; unpaired two-sided Student’s t-test; ****P < 0.0001. **D** Immunofluorescence of DMRT1^+^DDX4^+^ cells in monkey cultured SPG. The right panel shows the clone enlarged in the white dashed box of left field. Scale bars: 100 μm (left) and 10 μm (right). **E** Quantitative analysis of the number of DMRT1^+^DDX4^+^ cells in each group. Each circle represents the statistical result from one field of view (8 views from each group; *n* = 2 biologically independent samples). Data are presented as the mean ± SEM; unpaired two-sided Student’s t-test; ***P < 0.001. **F** Bright-field image of human SPG cells cultured with DMSO and EPZ. Scale bars: 500 μm. **G** Quantitative analysis of clone size for each group. Each circle represents the statistical result from one clone (12 clones from each group; *n* = 2 biologically independent samples). Data are presented as the mean ± SEM; unpaired two-sided Student’s t-test; **P < 0.01. **H** Immunofluorescence of UCHL1^+^ and DMRT1^+^ cells in human cultured SPG. The right panel shows the clone enlarged in the white dashed box of left field. Scale bars: 100 μm (left) and 10 μm (right). **I** and **J** Quantitative analysis of the number of UCHL1^+^ (**I**) and DMRT1^+^ (**J**) cells in each group. Each circle represents the statistical result from one field of view (8 views from each group; *n* = 2 biologically independent samples). Data are presented as the mean ± SEM; unpaired two-sided Student’s t-test; *P < 0.05, **P < 0.01

We then evaluated the effects of EPZ by culturing the sorted ITGA6^+^ cells from both species (Fig. 6A). In monkey cultures, EPZ treatment increased the number of UTF1^+^DDX4^+^ undifferentiated SPG while reducing the number of DMRT1^+^DDX4^+^ differentiating SPG compared with the DMSO control (Fig. 6B-E). In human cultures, the cell clones in the EPZ group were significantly larger than those in the DMSO group (Fig. 6F, G). A closer examination revealed that EPZ supplementation significantly increased the number of UCHL1^+^ undifferentiated SPG and decreased the number of DMRT1^+^ differentiating SPG, results consistent with a role in maintaining an undifferentiated SPG state (Fig. 6H-J).

To further assess these effects in a more physiological context, we cultured human testicular tissues with EPZ or vehicle. After 14 days of treatment, cultured human testicular tissues (donors with obstructive azoospermia [OA]) were collected to evaluate the number of germ cells, including undifferentiated SPG (Fig. S5C). We found that the percentage of human UTF1^+^ undifferentiated SPG in the lumen was higher in the EPZ group than in the vehicle group (Fig. S5D). In summary, our findings suggest that the PRMT5 inhibitor EPZ could exert conserved effects, supporting the undifferentiated state of SPG while suppressing their differentiation in mice, monkeys and humans.

## Discussions

The maintenance of human SPG in vitro is a long-standing question in the field. Although research strategies have begun to target differentiation-related pathways and relevant factors, the potential of epigenetic modifiers, as core regulators of cell fate, to establish a human SPG culture system remains largely unexplored. Here, we identified several epigenetic modulators that were upregulated during SPG differentiation. Given that small-molecule compounds provide a controllable and convenient strategy for manipulating cell fate (Chou And Cheng 2013; Hou et al. 2013), we performed a screen targeting these modulators, and found that PRMT5 inhibitor EPZ015666 significantly promoted the expansion of cultured mouse SSCs in vitro. Therefore, targeting inhibition of epigenetic factors upregulated during spermatogonial differentiation represents a novel culture strategy for maintaining undifferentiated SPG in vitro. However, whether this increase stems from a direct effect on SSC proliferation or is a phenotypic consequence of blocking differentiation requires further mechanistic elucidation.

A critical barrier to the long-term culture of primate SPG may lie in their inherent tendency under existing conditions to enter a differentiated spermatogonial state rather than maintaining an undifferentiated state (Bi et al. 2024). Interestingly, in both human and non-human primate testes, PRMT5 is also highly expressed in differentiated SPG (Güne And Kula 2013; Tan et al. 2020). Further experiments investigating the effects of PRMT5 inhibition on SPG in vitro demonstrated that the small molecule EPZ significantly increased the proportion of undifferentiated SPG in cultured human and monkey SPG. These findings suggest that EPZ effectively suppresses the differentiation tendency of SPG in vitro and maintains its undifferentiated state. This approach holds potential for maintaining the in vitro culture of SPG in both humans and non-human primates.

Previous studies have demonstrated that repopulation potential is not restricted to a specific subset of SSCs but is instead broadly and equally distributed across a large population of transplanted cells. Transient inhibition of spermatogonial differentiation has been shown to restore fertility in mice, thereby strengthening the model hypothesis (Nakamura et al. 2021). Consistently, we also found that EPZ treatment effectively inhibited spermatogonial differentiation and preserved a greater number of undifferentiated SPG under culture conditions that are prone to differentiation due to GDNF deprivation. For the fate of SPG (Hara et al. 2014; Klein et al. 2010), as it does not alter or compromise the intrinsic identity of the spermatogonia, nor does it impair their post-transplantation colonization capacity or subsequent spermatogenic potential. Building on this, our work establishes a potential strategy: the use of EPZ to simultaneously block differentiation and preserve the number of undifferentiated SPG in vitro, aiming to enhance cellular engraftment and spermatogenesis following transplantation. Therefore, the small molecule EPZ could hold promise for in vitro expansion of SPG and future clinical transplantation applications.

In postnatal male testicular tissue, PRMT5 has been shown to play an indispensable role in germ cell development and function. Specifically, previous studies indicate that postnatal knockout of *Prmt5* in male germ cells using *Stra8-Cre* leads to meiotic defects and male infertility (Wang et al. 2015), whereas depletion of *Prmt5* in mouse SSCs via *Ddx4-Cre* reduces *Plzf* expression, impairs self-renewal, and results in the gradual loss of SSCs (Dong et al. 2021). Importantly, the small molecule EPZ inhibits the enzymatic activity of PRMT5 without affecting its protein expression (Chan-Penebre et al. 2015). This characteristic supports the conclusion that EPZ treatment does not interfere with PRMT5 expression in cultured mouse SSCs or primate SPG. In line with this, our findings demonstrate that the differentiation-inhibitory effect of EPZ on spermatogonia stems specifically from its suppression of PRMT5 enzymatic activity. Notably, the precise molecular mechanism by which PRMT5 regulates differentiation through its methyltransferase activity remains unclear and warrants further investigation.

## Materials and methods

### Animal models

Mouse experiments were performed at the Animal Experimental Center of Southern Medical University, with the approval for animal studies from the competent institution. Mice were housed under a 12-h light/dark cycle, with temperature controlled between 20–25 °C and humidity maintained at 40%–70%. Mice had free access to water and food. *Prmt5 *^*flox/flox*^ mice were obtained from Institute of Zoology, Chinese Academy of Sciences (Bezzi et al. 2013; Dong et al. 2021). *Prmt5 *^*flox/flox*^; *Rosa26-CreERT2* mice were generated by crossing *Rosa26-CreERT2* mice with *Prmt5 *^*flox/flox*^ mice. The testicular tissue of Cynomolgus monkeys (*Macaca fascicularis*) was procured from Guangdong Institute of Biological Resources Application, which holds appropriate approvals for animal studies.

### Mouse SSC cell line establishment

The SSC cell line was established from 5.5 days postpartum (dpp) male B6D2F1 mice (EGFP-C57BL/6 × DBA/2). Cells were cultured according to a previously published protocol (Kanatsu-Shinohara et al. 2003): StemPro-34 SFM (Invitrogen) was supplemented with 1% FBS, 0.1 mM NEAA, 1 mM sodium pyruvate, 2 mM GlutaMAX, 100 U/mL penicillin, 0.1 mg/mL streptomycin, 50 μM β-mercaptoethanol, 1 μL/mL DL-lactic acid, 1 × minimal essential medium (MEM) Vitamin Solution, 10 μg/mL biotin, 100 μM ascorbic acid, 60 ng/mL progesterone, 30 ng/mL β-estradiol, 6 mg/mL Glucose, 5 mg/mL Bovine Serum Albumin (BSA), 1% N2 supplement, rat GDNF (512-GF, R&D Systems), 10 ng/mL recombinant mouse EGF (2028-EG, R&D Systems), 1000 U/mL recombinant mouse LIF (ESG1107, Millipore), and 10 ng/mL recombinant mouse FGF2 (3139-FB, R&D Systems).

For experimental groups: The EPZ group was cultured in medium where GDNF was replaced with 1 μM EPZ015666 (T6076, TargetMol, USA). The NTC group was cultured in medium where GDNF was replaced with DMSO (at an equal volume, serving as the vehicle control). The culture medium was changed every other day, and cells were passaged weekly at a split ratio of 1:3 to 1:5.

### Chemical library screening

Chemical libraries (listed in Table 1) were screened at a concentration of 10 µM using 96-well plates seeded with mitomycin C (MMC)-treated CF1 mouse embryonic fibroblast (MEF) feeder cells. Each plate included negative control wells (culture medium without GDNF) and compound-treated wells, with three replicates per condition. Ten thousand GFP-labeled cultured mouse SSCs were plated per well. After six days of culture, GFP fluorescence was quantified using a Cytation 5 imaging reader. The “clone number” metric was derived from GFP fluorescence measurements. Compounds that increased proliferation were identified based on a fold‑change threshold exceeding three standard deviations (3 SD) above the mean of the negative controls.

### Growth curve

A total of 300,000 cultured mouse SSCs were plated per well into a 12‑well plate pre‑seeded with feeder layer cells. Cells were maintained in either EPZ‑supplemented medium or NTC medium, with medium refreshed every two days. On day 6, cells were harvested, counted, and 300,000 viable cells were re‑plated into a new feeder‑coated well for the next passage. This process was repeated, with cell counts performed on day 6 of each passage. The cell proliferation curve was calculated based on this iterative process.

### Immunofluorescence of SSC clones

After clone formation, cells cultured on glass slides were fixed with 4% paraformaldehyde. The samples were permeabilized with PBS containing 0.5% Triton X-100 for 20 min and blocked with 2% BSA for 1 h at room temperature. Subsequently, samples were incubated with primary antibody overnight at 4 °C. Following three washes with PBS, they were incubated with appropriate fluorescently labeled secondary antibodies and Hoechst 33342 for 1 h at room temperature. After an additional three washes with PBS, slides were mounted using an anti‑fade mounting medium. Images were acquired using a confocal microscope (Carl Zeiss) and processed for further analysis.

### Paraffin-embedded sectioning of testicular tissue

Testicular tissue samples were washed twice with PBS and subsequently fixed in 4% paraformaldehyde overnight at 4 °C. After fixation, tissues were transferred to embedding cassettes and dehydrated through a graded ethanol series (70%, 80%, 90%, and 100%). Subsequently, the samples were cleared in xylene, infiltrated with paraffin, and embedded into paraffin blocks. Sections of 5–7 μm thickness were cut from the blocks and mounted on glass slides for subsequent analyses.

### Immunofluorescence of testicular tissue

Paraffin-embedded testicular tissue sections were deparaffinized in xylene and rehydrated through a graded ethanol series (100%, 90%, 80%, and 70%). For antigen retrieval, the sections were heated in EDTA buffer (pH 8.0) at 95 °C for 10 min and then allowed to cool at room temperature for 30 min. Subsequently, the sections were blocked with 2% BSA for 1 h at room temperature. Following blocking, the sections were incubated with the primary antibody overnight at 4 °C. After three washes with PBS, the sections were incubated with the corresponding fluorescently labeled secondary antibody and Hoechst 33,342 for 1 h at room temperature. The sections were then washed three times with PBS. Finally, the sections were mounted with an anti-fade mounting medium. Images were acquired using a confocal microscope (Carl Zeiss).

### In vivo transplantation of mouse SSCs and subsequent intracytoplasmic sperm injection

Male recipient pups were generated from timed-pregnant ICR mice. To deplete endogenous germ cells, pregnant dams received a single intraperitoneal injection of busulfan (40 mg/kg) at 12.5 days post coitum. Newborn male pups were used as transplant recipients. For transplantation, a glass needle was loaded with a mouse SSC suspension. Under microscopic guidance, the efferent ducts were identified, and the needle was inserted into a duct and advanced toward the rete testis. The cell suspension was gently expelled to fill the seminiferous tubules. The testis was carefully returned to the abdominal cavity after injection. The abdominal wall and skin were sutured, and the surgical site was cleaned. Pups were placed on a 37 °C warming pad until recovery before being returned to the dam.

Two months post-transplantation, testes were harvested and digested into a single-cell suspension. Cells were stained with Hoechst 33,342 for 20 min and GFP-positive round spermatids were isolated by flow cytometry (Beckman Coulter). The sorted spermatids were kept at 4 °C and used for round spermatid injection (ROSI) following established protocols.

### Culture of mouse testicular tissue

Testes from 6-day-postpartum (dpp) mice were dissected and cut into fragments of approximately 2 × 2 mm. The tissue fragments were placed on agarose blocks for air–liquid interface culture. Cultures were maintained in αMEM basal medium supplemented with 10% knockout serum replacement (KSR). For the experimental groups, 1 μM EPZ was added to the medium, while the control groups received an equivalent volume of DMSO. Samples were collected on culture days 7 and 21, fixed, and processed for paraffin sectioning and subsequent staining.

### In vivo validation of EPZ in mice

Male C57BL/6 J mice (8 weeks old; n = 3 per group) received a single oral gavage dose of EPZ015666 (10 mg/kg), formulated in 20% *N–N*-dimethylacetamide in water (Chan-Penebre et al. 2015). A control group received an equal volume of the vehicle solvent. On day 5 post-treatment, mice were anesthetized, euthanized, and testicular tissues were harvested and fixed.

### Evaluation of EPZ restorative effects following chemotherapy-Induced injury

To assess the restorative effect of EPZ015666 on SSCs after busulfan-induced depletion, adult male C57BL/6 J mice (8 weeks old) were used. Mice received a single intraperitoneal injection of busulfan (10 mg/kg; n = 3). On day 10 post-busulfan injection, mice were treated with either 10 mg/kg EPZ015666 or an equal volume of vehicle control via oral gavage. All mice were anesthetized and euthanized on day 20 post-busulfan injection, and testicular tissues were collected for fixation.

### Evaluation of EPZ protective effects during chemotherapy

To evaluate the protective effect of EPZ against chemotherapy-induced stress, mice were simultaneously administered a single intraperitoneal injection of busulfan (10 mg/kg) and either 10 mg/kg EPZ015666 or vehicle control via oral gavage (n = 3 per group). Ten days later, mice were anesthetized, euthanized, and testicular tissues were harvested for fixation.

### Human and monkey testicular sample preparation and cryopreservation

Human and monkey testicular tissue samples were immediately cut into smaller portions and cryopreserved using freezing media composed of 10% DMSO +40% αMEM +50% FBS under controlled cooling conditions in a freezing container at −80 °C. The samples were subsequently transferred to liquid nitrogen storage until use.

### Human and monkey SPG culture

Testicular tissue was mechanically disrupted and enzymatically digested with 1 mg/mL collagenase type IV at 37 °C for 2 h. Digestion was stopped by adding the same volume of αMEM +10% FBS medium, and the cells were size-filtered through 40 μm strainers and pelleted by centrifugation at 300 g for 5 min.

ITGA6^+^ cells were purified from dissociated testicular cells using the magnetic- activated cell sorting (MACS) system. Cells were resuspended in MACS buffer (PBS, 0.5% BSA and 2 mM EDTA) and incubated on ice with an anti-ITGA6 antibody (555,734, BD Pharmingen) for 30 min. After washing, cells were incubated with anti-rat IgG microbeads for 30 min on ice, washed in MACS buffer, re-suspended in 500 µL MACS buffer, and run through a MACS MS column (Miltenyi Biotec). Cells were then eluted with MACS buffer and resuspended in culture medium.

The medium for human and monkey SPG culture was as described in previously research (Tan et al. 2020) with some modifications: Iscove modified Eagle medium (IMDM) supplemented with 1 × ITS-X, 1 mM sodium pyruvate, 6 mg/mL Glucose, 1 μL/mL DL-lactic acid, 5 mg/mL BSA, 2 mM GlutaMAX, 50 μM β-mercaptoethanol, 1 × MEM vitamin solution, 0.1 mM NEAA, 100 µM ascorbic acid, 10 μg/mL d-Biotin, 30 ng/mL β-estradiol, 60 ng/mL progesterone, 0.2% chemically defined lipid concentrate (L0288, Sigma), 50 μl/mL KSR, 10 ng/mL recombinant human FGF2 (233-FB, R&D Systems), 15 ng/ml recombinant human GDNF (212-GD, R&D Systems), 10 ng/ml human recombinant BMP-8b (9316-BP, R&D Systems), 100 nM of the AKT1/2/3 inhibitor MK-2206 2HCl (S1078, Selleck Chemicals) and 1 μM EPZ. An equal volume of DMSO was used as the vehicle control in place of EPZ.

### Human seminiferous tubule in vitro culture

Biopsies of human testicular tissue samples were obtained and washed twice with PBS. Dissociate the sample into 2 mm pieces with a tweezer, place it on 1% agarose gel, then culture with SSC culture medium. In detail, StemPro-34 SFM with its supplement and 1% KSR was added. Supplied with 0.1 mM NEAA, 1 mM sodium pyruvate, 2 mM GlutaMAX, 100 U/mL penicillin, 0.1 mg/mL streptomycin, 50 μM β-mercaptoethanol, 1 μL/mL DL-lactic acid, 1 × minimal essential medium (MEM) Vitamin Solution, 10 μg/mL biotin, 100 μM ascorbic acid, 60 ng/mL progesterone, 30 ng/mL β-estradiol, 6 mg/mL Glucose, 5 mg/mL BSA, 1% N2 supplement, 20 ng/mL recombinant human GDNF and 10 ng/mL recombinant human FGF2. 1 μM EPZ015666 or DMSO solvent was added in necessary. Incubate the sample at 34 °C and change the medium every other day.

### CRISPR-Cas13d mediated *Prmt5* knockdown

The knockdown of *Prmt5* was conducted by using CRISPR-Cas13d system. Two sgRNAs designed to target the coding sequence of *Prmt5* mRNA were prepared:

- sg*Prmt*5-1F AAACAGACGCATTCACAAGAGGACCCC
- sg*Prmt*5-1R AAAAGGGGTCCTCTTGTGAATGCGTCT
- sg*Prmt5*−2F AAACGACGCATTCACAAGAGGACCCCG
- sg*Prmt5*−2R AAAACGGGGTCCTCTTGTGAATGCGTC

Non-targeting sgRNA (sgNT) for control was used as previously described. According to previous reports, lentivirus packaging and transfection were performed.

### Cell transfection

All siRNA transfections were performed using LipofectamineTM 3000 (11668019, Invitrogen) according to the manufacturer’s instructions. Briefly, Lipofectamine 3000 reagent and siRNAs were separately diluted in Opti-MEM medium (31985070, GIBCO). Then, the diluted siRNAs were added to the diluted Lipofectamine 3000 reagent, followed by 20 min incubation at room temperature. Subsequently, the mixtures were added to the cell suspension. After 60 h, the cells were collected and analyzed. Two siRNAs designed to target the coding sequence of *Prmt5* mRNA were prepared:

- siRNA-1: GCCACCACTCTTCCATGTT
- siRNA-2: GCACAGTTTGAGATGCCTT

### Western blot

Cells were lysed in RIPA Lysis buffer (R0010, Solarbio) to collect the cellular proteins, which were resolved by SDS-PAGE and transferred to PVDF membrane (RPN303F, GE). The membrane was blocked and then incubated overnight with primary antibodies against PRMT5 (18436–1-AP, Proteintech) and DMRT1 (sc-377167, Santa Cruz Biotechnology). Immuno-reactive bands were visualized using the ECL Western Blotting Substrate Kit (36208ES60, YEASON) before exposure. The intensities of bands were quantified with ImageJ (http://imagej.nih.gov/ij/).

### Quantitative real-time PCR analysis

SSC samples were collected after 18 days of culture under different conditions, and total RNA was extracted using TRIzol reagent (DP424, TIANGEN) according to the manufacturer’s instructions. RNA was subsequently reverse transcribed to cDNA using the HiScript Q RT SuperMix Kit (R123-01, Vazyme). Then, cDNA was diluted and used for RT-qPCR with RealStar Green Fast Mixture reagent (A301-101, GenStar) and designed primers on a LightCycler®96 (900012C, Roche). Each sample was run in triplicate. Relative mRNA expression levels of targeted genes were calculated through the 2-ΔΔCT method after being normalized by housekeeping gene Gapdh. The detailed sequence of primers was listed.

Gene name Forward primer sequence Reverse primer sequence.

*Gapdh *TGTGTCCGTCGTGGATCTGA TTGCTGTTGAAGTCGCAGGA.

*Kit *CTCCCCCAACAGTGTATTCACTAGCCCGAAATCGCAAATCTT.

*Dmrt1 *GCCACGTCTCAGCCATCTG GTCGCCAGCTTCAACTATTAACT.

*Stat3 *CAATACCATTGACCTGCCGATGAGCGACTCAAACTGCCCT.

### Data acquisition and preprocessing

Single-cell RNA-seq datasets of human and rhesus macaque testes were collected from the Gene Expression Omnibus (GEO) under accession number GSE142585 (Shami et al. 2020). Mouse testicular single-cell transcriptomic data were obtained from GSE112393 (Green et al. 2018). These datasets were integrated and analyzed for comparative characterization of Spermatogonia across species. Orthologous genes were identified using the R package HomoloGene (v1.4.68). Pairwise orthologues (human vs macaque, human vs mouse) are retrieved separately and merged using human Ensembl gene ID as reference. Orthologues common to all three species were used for cross-species comparative analysis. Spermatogonia from the three species were analyzed jointly. Only cells expressing more than 600 genes and with less than 10% mitochondrial reads were retained for further analysis.

### Dimension reduction and cell-type annotation

We utilized the R package Seurat (v4.4.0) for comprehensive single-cell analysis. The global-scaling normalization method ‘LogNormalize’ was applied for data normalization. The functions ‘FindIntegrationAnchors’ and ‘IntegrateData’ were used to integrate different batches of scRNA-seq data. Based on the ‘JackStraw’ results, the top 15 principal components were selected to construct a K‑nearest neighbor (KNN) graph and to perform nonlinear dimensionality reduction (t‑SNE). Cell clustering was carried out using the ‘FindClusters’ function with a resolution parameter set to 0.2. Each cluster was subsequently annotated with a cell type based on known marker genes.

### Gene score calculation

Gene scores for each cell were calculated using the ‘AddModuleScore’ function from Seurat with default parameters. The gene sets were obtained from the Molecular Signatures Database (https://www.gsea-msigdb.org/gsea/msigdb).

### RNA-seq library construction and sequencing

For mRNA library preparation, 1 µg of total RNA per sample was used. The mRNA was then fragmented, followed by cDNA synthesis and purification. The purified cDNA was subjected to end repair, A-tailing, and adapter ligation. cDNA fragments of 200–300 bp were selected, PCR-amplified, and purified to generate the final library. Library quality was assessed using the Agilent 4200 TapeStation. Sequencing was carried out on an Illumina NovaSeq platform with PE150 configuration.

### RNA-seq data analysis

Raw reads were processed with BBmap (v39.01) and Trimmomatic (v0.39) to remove adapter sequences and low-quality bases. The resulting clean reads were aligned to the GRCm38 reference genome (Ensembl) using HISAT2 (v2.2.1). Gene expression levels were quantified with StringTie (v2.2.1).

### Weighted correlation network analysis

Weighted gene co‑expression network analysis was performed using the R package WGCNA (v1.7.3) (Langfelder And Horvath 2008). A scale-free topology (R^2^ > 0.80) was achieved through dynamic soft‑thresholding. Gene modules were identified via topological overlap matrix (TOM)‑based clustering, with a minimum module size of 70 and a merge cut‑height of 0.3. Module–trait correlations were calculated using Pearson correlation coefficients.

### Statistical analysis

Microsoft Excel, GraphPad Prism (version 8.0.1) and R (version 3.5.1) were used for statistical calculations. Specific statistical tests, sample number and other information are indicated in the main text or figure legends. The t-test and two-sided statistical analysis approach were used to determine the significance of differences between sets of data if there was no specific indication. All experiments were conducted at least twice independently, and similar results were adopted for further analysis to guarantee reproducibility.

## Supplementary Information

Supplementary Material 1: Figure S1. Merged analysis of mouse, human, and macaque SPG, Related to Fig. 1. (A) t-SNE plots of SPG from mice, monkeys and humans. Left: cells colored by species. Right: cells colored by the identified cell types. (B) Gene expression patterns of marker genes on t-SNE plots. A gradient of gray to red indicates low to high expression levels. (C) Dot plot showing representative marker genes across cell clusters. Dot size is proportional to the fraction of cells expressing specific genes. Color intensity corresponds to the relative expression of specific genes. (D) Violin plot showing gene set scores across cell subtypes. Statistical significance was assessed using the Wilcoxon test. Figure S2. Evaluation of EPZ Efficacy in Mice, Related to Fig. 2. (A) Schematic outline of EPZ injection under physiological conditions. (B) Immunofluorescence of SOX9 co-stained with PLZF in a section of mouse testis on day 6 after injection (up). Quantitative analysis of the percentage of PLZF^+^ cells among SOX9^+^ cells for each group (down). Data are presented as the mean ± SEM (*n* = 3 biologically independent samples); unpaired two-sided Student’s t-test; *P < 0.05. Scale bar, 50 μm. (C) Immunofluorescence of KIT co-stained with PLZF in a section of mouse testis on day 6 after injection (up). Quantitative analysis of the percentage of KIT^+^ cells (down). Data are presented as the mean ± SEM (*n* = 3 biologically independent samples); unpaired two-sided Student’s t-test; *P < 0.05. Scale bar, 50 μm. Figure S3. Mouse testicular tissue culture and SSC transplantation, Related to Fig. 3. (A) Schematic for the 6 dpp mouse testis organ culture. Mouse testes were treated with DMSO or EPZ at day 7. (B) Immunofluorescence of DDX4^+^ID4^+^ cells in a section of mouse testis after in vitro culture at day 7. Scale bar, 20 μm. (C) Quantitative analysis of the percentage of ID4^+^ SSCs among DDX4^+^ cells in each group. Each circle represents the statistical result from one tissue Sect. (16 sections from each group; *n* = 3 biologically independent samples). Data are presented as the mean ± SEM; unpaired two-sided Student’s t-test; **P < 0.01. (D) Immunofluorescence of STRA8^+^DDX4^+^ cells in a section of mouse testis after in vitro culture at day 7. Scale bar, 20 μm. (E) Quantitative analysis of the percentage of STRA8^+^ cells among DDX4^+^ cells in each group. Each circle represents the statistical result from one tissue Sect. (11 sections from each group; *n* = 3 biologically independent samples). Data are presented as the mean ± SEM; unpaired two-sided Student’s t-test; ****P < 0.0001. (F) Immunofluorescent staining of PLZF^+^ cells in recipient testes that were transplanted with GFP-labeled SSCs with EPZ-treatment after 10 passages. Arrows indicate PLZF^+^ cells. Scale bar, 20 μm. (G) Statistics for embryo development and the generation of offspring from spermatids. Figure S4. *Prmt5* knockdown suppresses spermatogonial differentiation, Related to Fig. 5. (A) The expression of PRMT5 and DMRT1 was analyzed by western blotting in cultured mouse SSCs. (B) Quantitative analysis of relative PRMT5 protein level in (A) from three independent experiments. Error bars indicate mean ± SEM; unpaired two-sided Student’s t-test; *P < 0.05, **P < 0.01. (C) Quantitative analysis of relative DMRT1 protein level in (A) from three independent experiments. Error bars indicate mean ± SEM; unpaired two-sided Student’s t-test; **P < 0.01, ****P < 0.0001. (D) qPCR analysis of relative expression levels from *Prmt5* knockdown cells mediated by the CRISPR-Cas13d system. Error bars indicate mean ± SEM from three independent experiments; unpaired two-sided Student’s t-test; ns, non-significant, *P < 0.05, **P < 0.01, ***P < 0.001. Figure S5. Isolation of SPGs and culture of human testicular tissue, Related to Fig. 6. (A) Immunofluorescence of UTF1^+^DDX4^+^ cells was performed on ITGA6^+^ and ITGA6^−^ cells from monkey testicular cells by MACS (left). Scale bar, 50 μm. Quantitative analysis of the percentages of UTF1^+^DDX4^+^ cells for each group (right). Each circle represents the statistical result from one field of view (6 views from each group; *n* = 2 biologically independent samples). Data are presented as the mean ± SEM; unpaired two-sided Student’s t-test; ****P < 0.0001. (B) Immunofluorescence of UCHL1^+^ID4^+^ cells was performed on ITGA6^+^ and ITGA6^−^ cells from human testicular cells by MACS (left). Scale bar, 50 μm. Quantitative analysis of the percentages of UCHL1^+^ID4^+^ cells in each group (right). Each circle represents the statistical result from one field of view (6 views from each group; *n* = 2 biologically independent samples). Data are presented as the mean ± SEM; unpaired two-sided Student’s t-test; ****P < 0.0001. (C) Schematic outline of human seminiferous tubule culture condition in vitro. (D) Immunofluorescent staining of UTF1^+^DDX4^+^ undifferentiated SPG in seminiferous tubule cultured tissue (left). Scale bars, 10 μm. Quantitative analysis of the percentages of UTF1^+^ cells in seminiferous tubules (right). Data are presented as the mean ± SEM (*n* = 2 biologically independent samples); unpaired two-sided Student’s t-test.

## Data Availability

All sequencing data generated during this study are available from the GEO under the accession number GSE319135.

## Abbreviations

SSCs: Spermatogonial stem cells
PRMT5: Protein arginine methyltransferase 5
GDNF: Glial cell line–derived neurotrophic factor
GSCs: Germline stem cells
SPG: Spermatogonia
FACS: Fluorescence-activated cell sorting
MACS: Magnetic-activated cell sorting
WGCNA: Weighted correlation network analysis
