# Supplementary material for: PRMT5 inhibition promotes cross-species spermatogonia expansion and suppresses differentiation
Source: Cell Regen. 2026 Jul 7;15:22. doi: 10.1186/s13619-026-00293-x (PMC13338090; doi:10.1186/s13619-026-00293-x)
Supplement: Supplementary file 1 — Supplementary Material 1: Figure S1. Merged analysis of mouse, human, and macaque SPG, Related to Fig. 1. (A) t-SNE plots of SPG from mice, monkeys and humans. Left: cells colored by species. Right: cells colored by the identified cell types. (B) Gene expression patterns of marker genes on t-SNE plots. A gradient of gray to red indicates low to high expression levels. (C) Dot plot showing representative marker genes across cell clusters. Dot size is proportional to the fraction of cells expressing specific genes. Color intensity corresponds to the relative expression of specific genes. (D) Violin plot showing gene set scores across cell subtypes. Statistical significance was assessed using the Wilcoxon test. Figure S2. Evaluation of EPZ Efficacy in Mice, Related to Fig. 2. (A) Schematic outline of EPZ injection under physiological conditions. (B) Immunofluorescence of SOX9 co-stained with PLZF in a section of mouse testis on day 6 after injection (up). Quantitative analysis of the percentage of PLZF+ cells among SOX9+ cells for each group (down). Data are presented as the mean ± SEM (n = 3 biologically independent samples); unpaired two-sided Student’s t-test; *P < 0.05. Scale bar, 50 μm. (C) Immunofluorescence of KIT co-stained with PLZF in a section of mouse testis on day 6 after injection (up). Quantitative analysis of the percentage of KIT+ cells (down). Data are presented as the mean ± SEM (n = 3 biologically independent samples); unpaired two-sided Student’s t-test; *P < 0.05. Scale bar, 50 μm. Figure S3. Mouse testicular tissue culture and SSC transplantation, Related to Fig. 3. (A) Schematic for the 6 dpp mouse testis organ culture. Mouse testes were treated with DMSO or EPZ at day 7. (B) Immunofluorescence of DDX4+ID4+ cells in a section of mouse testis after in vitro culture at day 7. Scale bar, 20 μm. (C) Quantitative analysis of the percentage of ID4+ SSCs among DDX4+ cells in each group. Each circle represents the statistical result from one tissue Sect [file 13619_2026_293_MOESM1_ESM.pdf]

Figure S1.

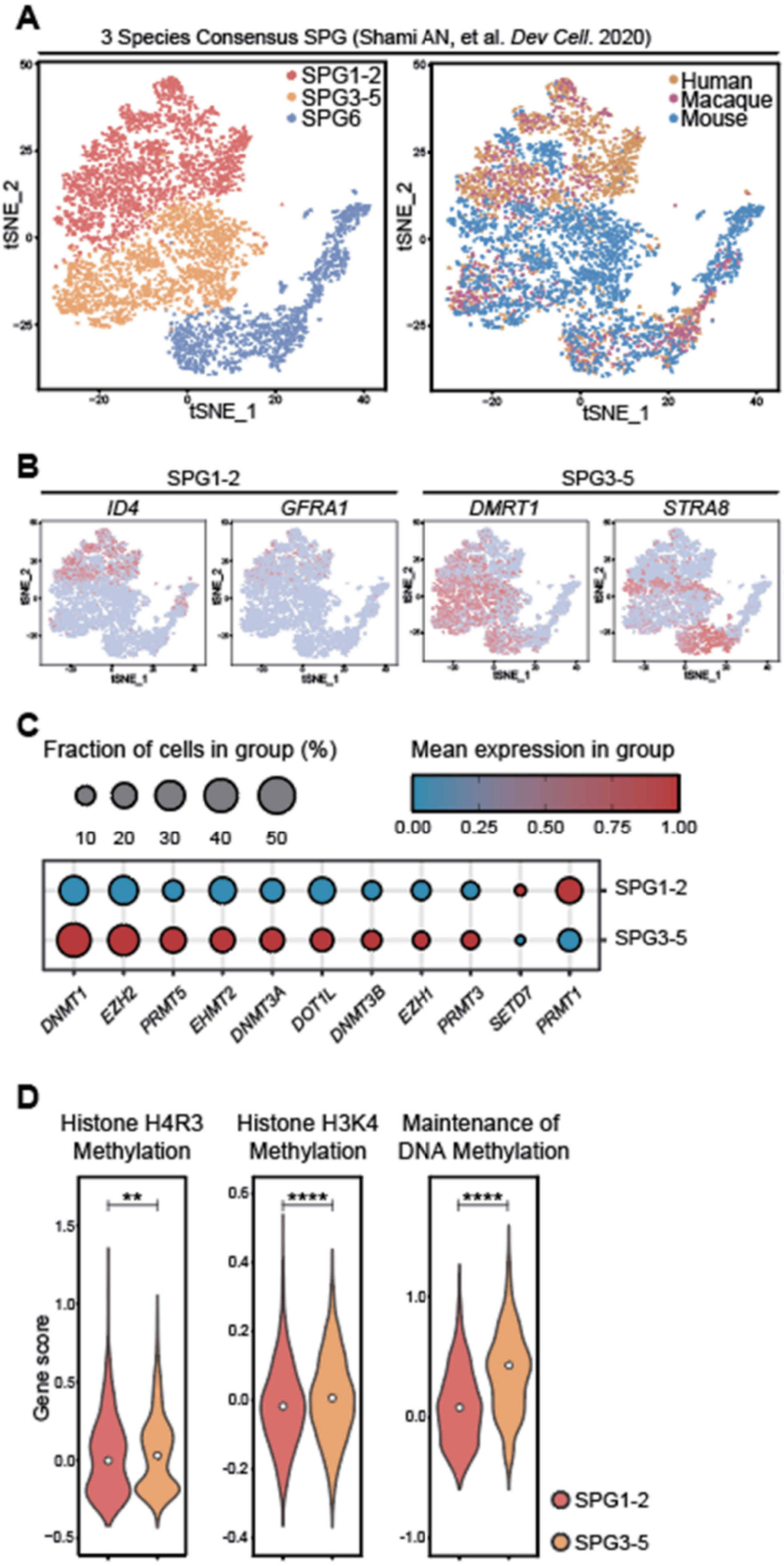
A
3 Species Consensus SPG (Shami AN, et al. Dev Cell. 2020)
SPG1-2
SPG3-5
SPG6
Human
Macaque
Mouse
tSNE_1
tSNE_2
B
SPG1-2
SPG3-5
SPG6
ID4
GFRA1
DMRT1
STRA8
SYCP2
HORMAD1
Expression
C
Fraction of cells in group (%)
10 20 30 40 50
Mean expression in group
0.00 0.25 0.50 0.75 1.00
SPG1-2
SPG3-5
DNMT1
EZH2
PRMT5
EHMT2
DNMT3A
DOT1L
DNMT3B
EZH1
PRMT3
SETD7
PRMT1
D
Histone H4R3 Methylation
Histone H3K4 Methylation
Maintenance of DNA Methylation
Gene score
**
****
****
SPG1-2
SPG3-5

Figure S2.

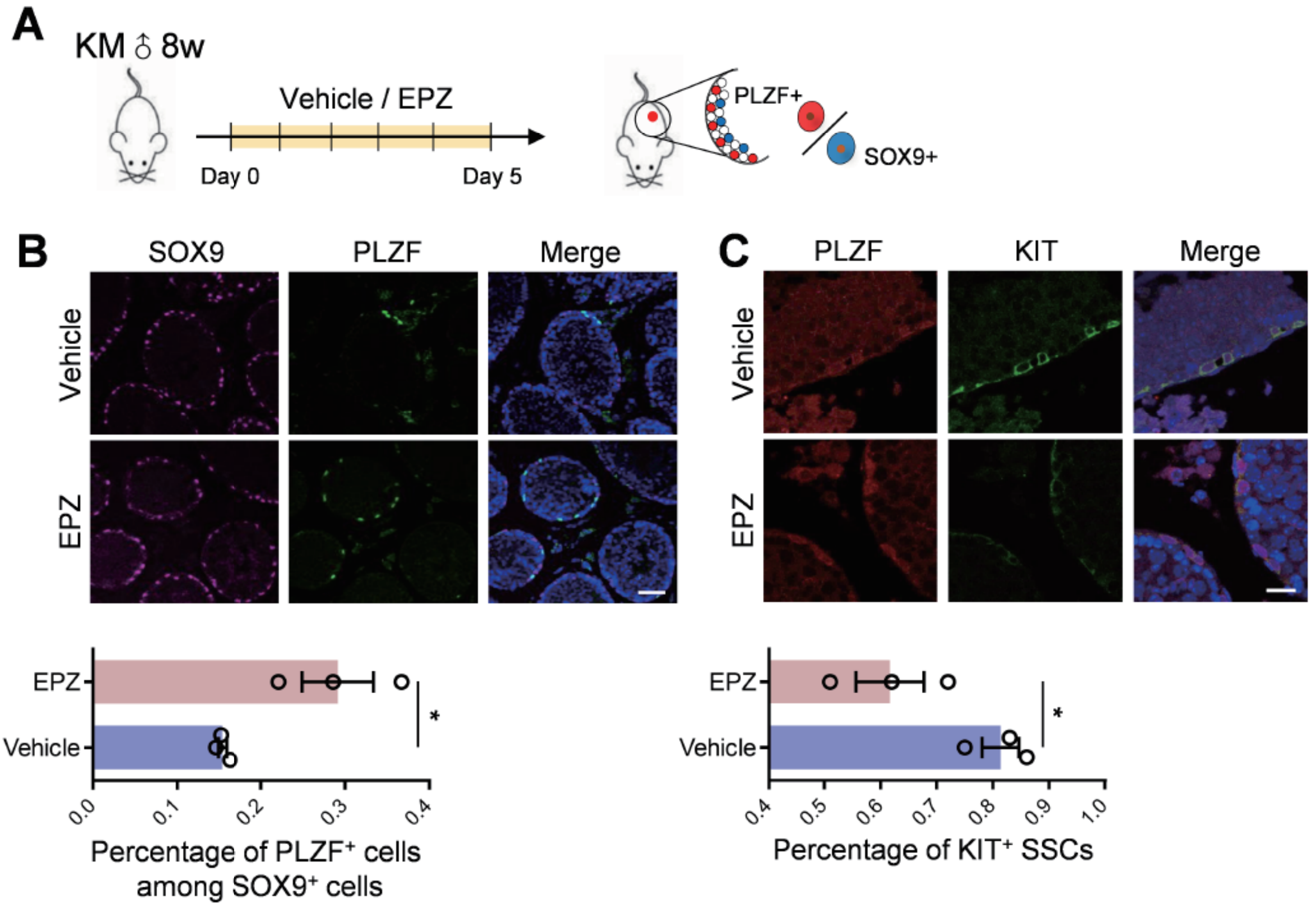

A
KM ♂ 8w
Vehicle / EPZ
Day 0
Day 5
PLZF+
SOX9+
B
SOX9
PLZF
Merge
Vehicle
EPZ
EPZ
Vehicle
0.0
0.1
0.2
0.3
0.4
*
Percentage of PLZF⁺ cells among SOX9⁺ cells
C
PLZF
KIT
Merge
Vehicle
EPZ
EPZ
Vehicle
0.4
0.5
0.6
0.7
0.8
0.9
1.0
*
Percentage of KIT⁺ SSCs

Figure S3.

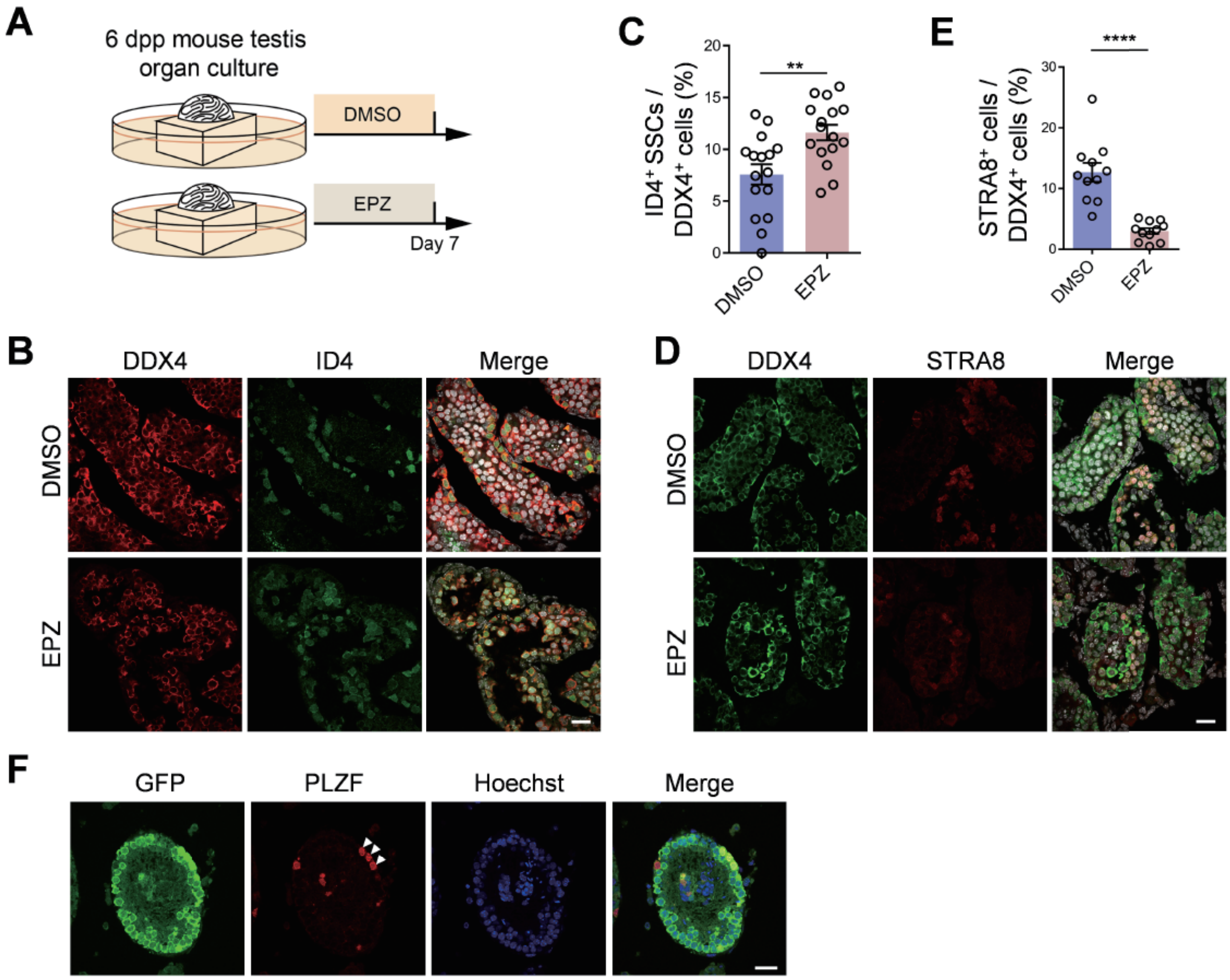

G

Origin of spermatids	No. of oocytes injected (n)	No. of 2-cell embryos (n)(%)	No. of 2-cell embryos transferred(n)	No. of pups survived (n)(%)
EPZ-treated SSCs	14	12 (85.7)	12	3 (25.0)
In vivo testis	20	20 (100)	20	6 (30.0)

Figure S4.

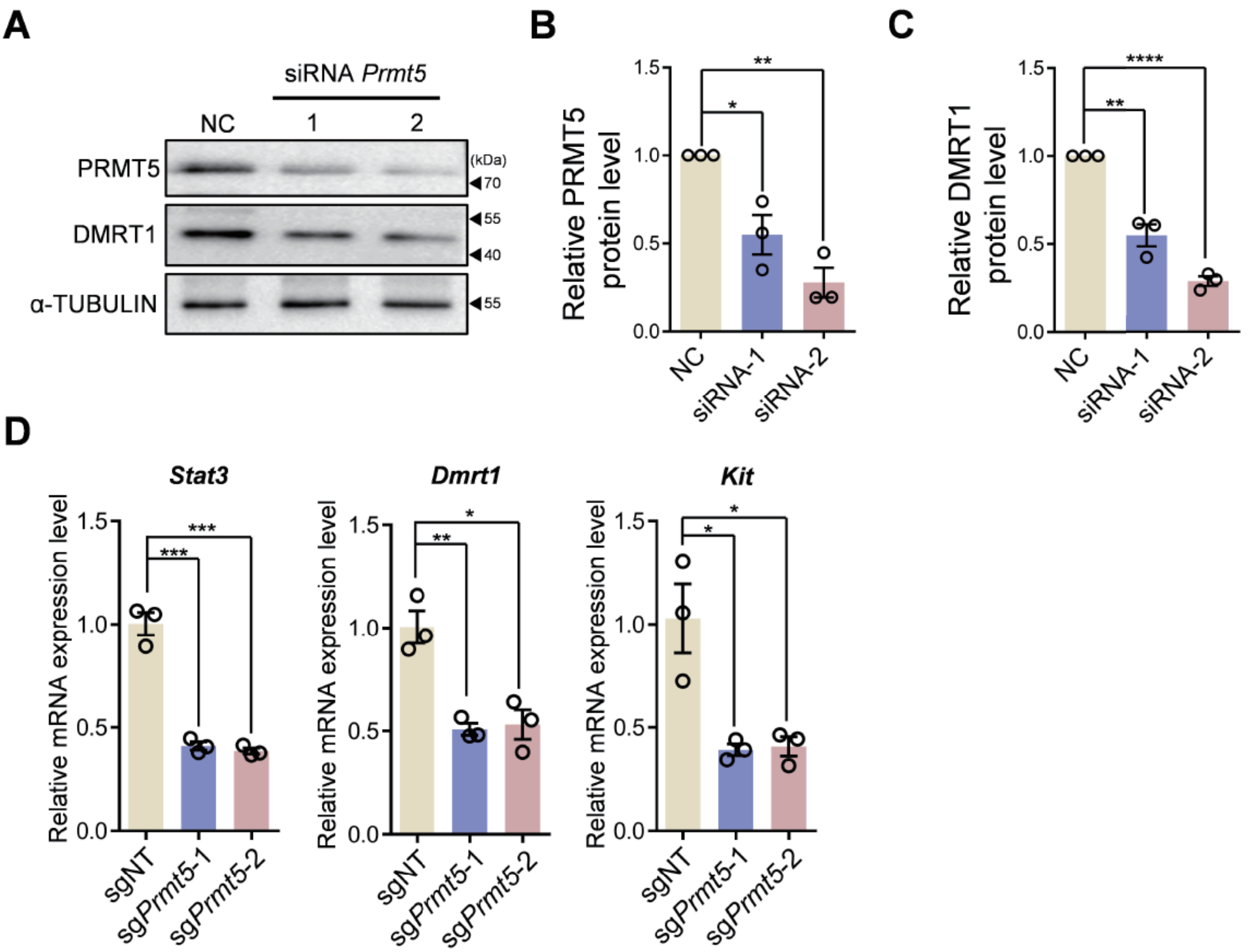
A
siRNA Prmt5
NC
1
2
PRMT5
DMRT1
α-TUBULIN
(kDa)
70
55
40
55
B
Relative PRMT5 protein level
NC
siRNA-1
siRNA-2
C
Relative DMRT1 protein level
NC
siRNA-1
siRNA-2
D
Stat3
Dmrt1
Kit
Relative mRNA expression level
sgNT
sgPrmt5-1
sgPrmt5-2

Figure S5.

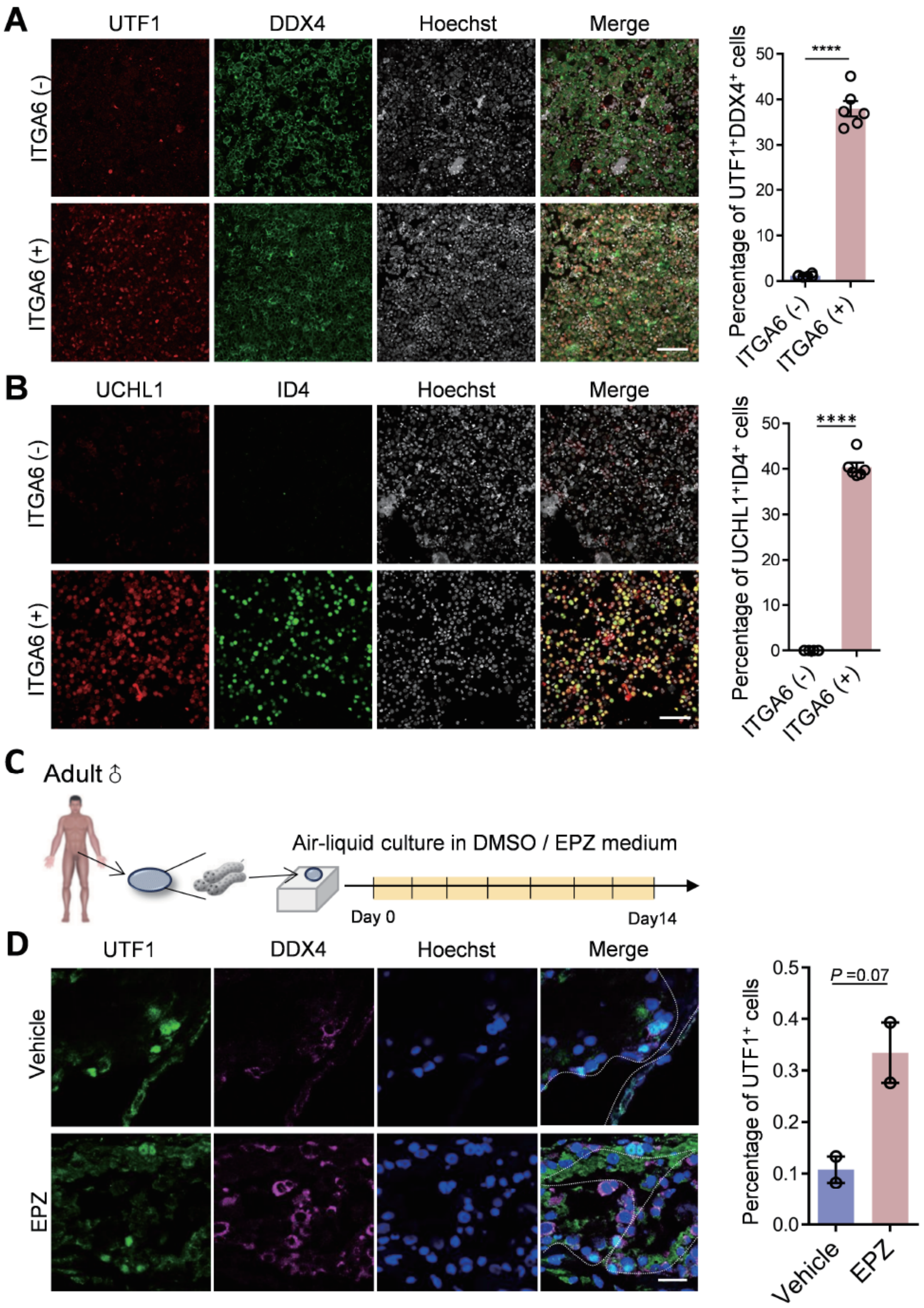
A
UTF1
DDX4
Hoechst
Merge
ITGA6 (-)
ITGA6 (+)
Percentage of UTF1⁺DDX4⁺ cells
****
50
40
30
20
10
0
B
UCHL1
ID4
Hoechst
Merge
ITGA6 (-)
ITGA6 (+)
Percentage of UCHL1⁺ID4⁺ cells
****
C
Adult ♂
Air-liquid culture in DMSO / EPZ medium
Day 0
Day14
D
UTF1
DDX4
Hoechst
Merge
Vehicle
EPZ
Percentage of UTF1⁺ cells
P =0.07
0.5
0.4
0.3
0.2
0.1
0.0
